# Allelic variation alters expression and antigen presentation of MR1 allomorphs

**DOI:** 10.1016/j.jbc.2026.111470

**Published:** 2026-04-17

**Authors:** Adam G. Nelson, Victoria Letoga, Clarice Z.Q. Lee, Carolyn Samer, Songyi Li, Lucy J. Meehan, Hamish E.G. McWilliam, Nicholas A. Gherardin, Allison Abendroth, Jose A. Villadangos, Barry Slobedman, Alexandra J. Corbett, James McCluskey, Jamie Rossjohn, Zhenjun Chen, Michael N.T. Souter, Wael Awad

**Affiliations:** 1 Department of Microbiology and Immunology, University of Melbourne at the Peter Doherty Institute for Infection and Immunity, Victoria, Australia; 2 Department of Biochemistry and Molecular Biology, Immunity Program, Biomedicine Discovery Institute, Monash University, Victoria, Australia; 3 Infection, Immunity and Inflammation, School of Medical Sciences, Faculty of Medicine and Health, and the Charles Perkins Centre and Sydney Institute of Infectious Diseases, The University of Sydney, Camperdown, New South Wales, Australia; 4 Department of Biochemistry and Pharmacology, Bio21 Molecular Science and Biotechnology Institute, University of Melbourne, Victoria, Australia; 5 Institute of Infection and Immunity, Cardiff University, School of Medicine, Cardiff, United Kingdom

**Keywords:** allele, antigen, MAIT, MR1, polymorphism

## Abstract

The major histocompatibility complex (MHC) class I-related protein 1 (MR1) presents vitamin B-derived metabolites to mucosal-associated invariant T (MAIT) and other T cells. There is limited polymorphism of MR1, the functional impact of which is not understood. We examined the impact of allelic variation of MR1 on the expression, structure and function of the known MR1 allomorphs. The expression and function of MR1∗02, MR1∗03, and MR1∗06 were similar to the canonical MR1∗01. Crystal structures of four MR1 allomorphs show that their polymorphisms do not impact the three-dimensional fold of MR1. Despite the binding of 5-OP-RU to MR1∗05 and its cell surface upregulation, this allomorph was severely impaired in its ability to activate primary MAIT cells. This phenotype was controlled by two (His90Gln and Glu52Gly) of its three polymorphisms, which led to the loss of structurally stabilizing interactions. When cells expressing the MR1 allomorphs were infected with herpes simplex virus type 1 (HSV-1), the nascent expression of all allomorphs was severely impaired, but surface expression of MR1∗04:01 and MR1∗04:02 was relatively less impacted. Hence, MR1 allelic variation alters the expression and function of the MR1∗04 and MR1∗05 allomorphs, with implications for MAIT cell and diverse MR1-reactive T cell immunity.

The major histocompatibility complex (MHC) class I-related protein 1 (MR1) contributes to immune surveillance by presenting small molecule metabolite antigens (Ag) to populations of MR1-reactive T cells, including mucosal-associated invariant T (MAIT) cells (1, 2, 3). MR1 and the MAIT cell T cell receptor (TCR) are highly conserved across most mammals suggesting an important function across species (4, 5, 6, 7, 8, 9, 10). MAIT cells are characterised by a semi-invariant TCR of a single α-chain paired with a limited pool of β-chains (10, 11), expression of the master transcription factor promyelocytic leukaemia zinc finger and expression of CD161 in humans (12, 13). MR1 has low basal surface expression in most cells and MR1 molecules are retained in the endoplasmic reticulum in a ligand-receptive conformation (14, 15). MR1 is upregulated in the presence of ligands that form a Schiff base with MR1-Lys43 within the antigen-binding pocket that induces transit to the plasma membrane to activate MAIT and other MR1-reactive T cells (14). To date, the most potent MAIT cell antigens are 5-(2-oxopropylideneamino)-6-D-ribitylaminouracil (5-OP-RU) and 5-(2-oxoethylideneamino)-6-D-ribitylaminouracil (5-OE-RU). These ligands are the nonenzymatic condensation products of 5-amino-6-(D-ribitylamino)-uracil (5-A-RU), a precursor of riboflavin synthesis in microbes, and methylglyoxal or glyoxal derived from host/microbe metabolism (1, 16). Microbial riboflavin metabolites, such as 5-OP-RU, are involved in MAIT cell development (17, 18). In addition to riboflavin metabolites, MR1 can also present diverse ligands including self-metabolites such as bile acids and nucleobase adducts, folate derivatives such as 6-formylpterin (6-FP) and acetylated 6-FP (Ac-6-FP), pyridoxine derivatives, drug and drug-like compounds, dietary, and environmental molecules (19, 20, 21, 22, 23, 24) to a diverse panel of MR1-reactive T cells (20, 25, 26, 27). This Ag diversity suggests that MR1 may be involved more broadly as an immune sensor of metabolism and/or stress (28). Furthermore, we have recently demonstrated that several herpesviruses negatively impact the antigen presentation of MR1 to MAIT cells, potentially implicating this molecule in anti-viral immunity (29, 30).

Unlike MHC-I, which is polygenic and highly polymorphic (31, 32, 33), MR1 is highly conserved in humans such that they generally share a single allele, MR1∗01 (8, 9, 34, 35). This is similar to the low level of polymorphism observed in the few studies of the *CD1*a-e genes where limited polymorphism is found mainly in exon 2 but in some isoforms in exon 3, with *CD1e* possessing six alleles. However, the functional significance of CD1 polymorphism hasn’t been fully determined (36), and several CD1 mutations are quite rare or predicted to be silent. HLA-E has two frequent alleles found worldwide, but systematic genomic sequencing has found numerous low-frequency polymorphisms, including many in non-coding and 3′UT regions (37). Thus, HLA-E polymorphism sits somewhere between the limited polymorphism of MR1 and CD1 and the extreme polymorphism of classical *HLA* genes.

Previously, we identified a patient with a rare non-synonymous polymorphism of Arg to His at position 9 (MR1^R9H^) of the antigen-binding cleft, associated clinically with persistent viral and bacterial infections that were difficult to treat (18). This MR1^R9H^ mutation abrogates antigen presentation of 5-OP-RU (18), resulting in the absence of MAIT cells, a defect that may impact the ability of this patient to control persistent skin infections (18). Five additional *MR1* alleles were recently identified by sequencing the protein-coding region of the MR1 gene from a small cohort of individuals with diverse HLA haplotypes (35). These alleles were numbered in order of their reported frequencies. MR1∗01 (71%), MR1∗02 (25%), MR1∗03 to 06 (∼1%). Each *MR1* allele contains non-synonymous mutations in the coding region predicted to modify the mature MR1 protein and potentially the function of MR1 (35). Indeed, we have recently discovered that MR1^R9H^ preferentially presents vitamin B6-related antigens to a subset of diverse MR1-reactive T cells (21). Further, the role of MR1^R9H^ in sensing cancer-enriched antigens has previously been reported (38, 39), but the broader impact of MR1 allelic mutations on MR1 function, MAIT immune responses and protective immunity against pathogens has not been described.

In this study, using cellular, biochemical and structural approaches, we investigated the effect of the known MR1 polymorphisms on antigen presentation, activation of MAIT and diverse MR1-reactive T cells, and suppression of MR1 expression by herpes simplex virus type 1 (HSV-1). We found that allomorphs MR1∗01, MR1∗02, MR1∗03 and MR1∗06 were comparable in their surface and intracellular expression, their binding of canonical MR1 ligands and their ability to activate primary MAIT cells. We demonstrate that MR1∗04 allomorphs bearing the MR1^R9H^ mutation had greater surface and intracellular expression, an altered ligandome and a differential capacity to activate diverse non-MAIT MR1-reactive T cells. In contrast, the MR1∗05 allomorph showed impaired steady state cell surface expression, poor stability and impaired Ag presentation when compared to the other allelic variants. Crystal structures of the different MR1 allomorphs showed that while the overall architecture of MR1 was not impacted, specific polymorphisms altered ligand repertoire and MR1 stability. Furthermore, we demonstrate that, as previously described in the case of MR1∗01, HSV-1 infection prevents cell surface expression of nascent MR1∗02, MR1∗03, MR1∗05 and MR1∗06. Expression of nascent MR1∗04 was also inhibited, but steady cell surface expression of these latter allomorphs was less impacted. Collectively, we provide a framework for understanding the impact of MR1 polymorphism in human immunity.

## Results

### Altered cell surface expression of MR1∗04:01, MR1∗04:02, and MR1∗05

**Figure 1:**
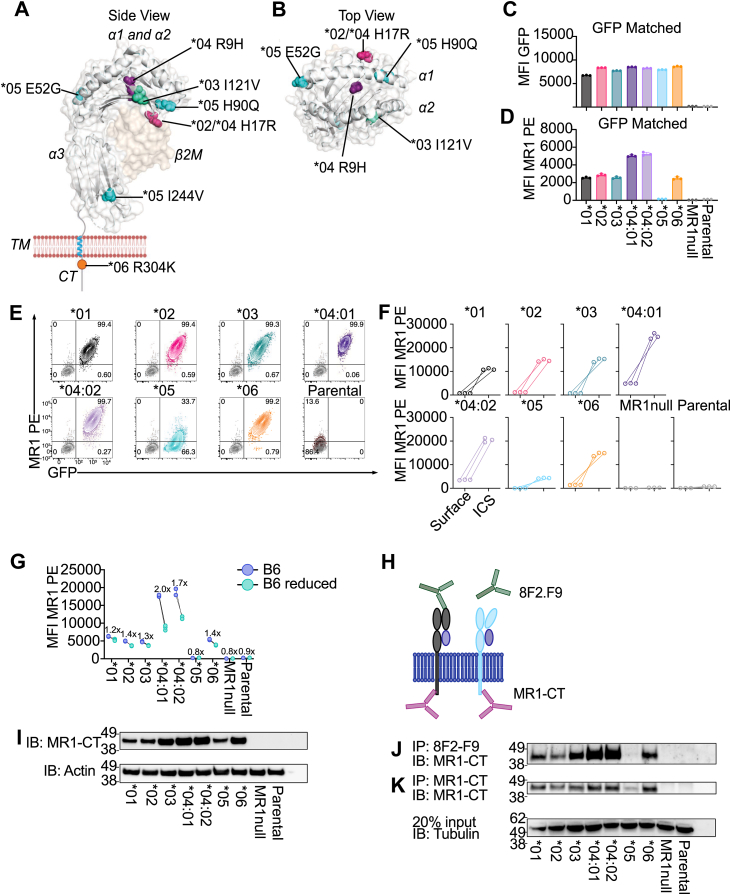
**Differential surface and intracellular expression of MR1 allomorphs.***A* and *B*, schematic of the side (*A*) and top (*B*) of MR1 (PDB:4GUP) highlighting each MR1 polymorphism, colour-coded based on allelic variant: MR1∗02/04:02 (His17Arg) *pink*, MR1∗03 (Ile121Val) *green*, MR1∗04:01/04:02 (Arg9His) *purple*, MR1∗05 (Glu52Gly, His90Gln and Ile244Val) *light blue*, and MR1∗06 (Arg304Lys) *orange*. *C* and *D*, constitutive expression of GFP (*C*) and median fluorescence intensity (MFI) from anti-MR1-PE surface staining (*D*) for MR1 allomorph cell lines matched for GFP/MR1 expression. *E*, representative flow cytometry panels showing C1R.MR1^null^ cell lines transduced with MR1∗01 (*black*), MR1∗02 (*pink*), MR1∗03 (*green*), MR1∗04:01 (*dark purple*), MR1∗04:02 (*light purple*), MR1∗05 (*blue*), MR1∗06 (*orange*) or C1R parental cells (*dark red*) compared to C1R.MR1^null^ cells (*grey*) for anti-MR1-PE surface staining (*y axis*) and GFP (*x axis*). Quadrant gates display percentage of live cells. *F*, paired graph showing surface MR1 expression compared to intracellular MR1 expression for each MR1 allomorph cell line. *G*, paired graph showing MR1 surface expression of MR1 allomorph cell lines cultured for 16 h in standard media that includes vitamin B6 supplement (*blue dots*, B6) or media with reduced vitamin B6 (*green dots*, B6 reduced). Annotation displays fold change between conditions. *H*, schematic depicting approximate binding sites of anti-MR1-CT and 8F2.F9 antibodies (*I*) Immunoblots (IB) from lysed MR1 allomorph cell lines with detection of MR1 using anti-MR1-CT or control actin. Axis numbers display protein ladder (kDa). *J* and *K*, immunoprecipitations (IP) from MR1 allomorph cell lysates pulled down with anti-MR1-CT (*J*) or 8F2.F9 (*K*) and immunoblotted for MR1 using anti-MR1-CT or control tubulin. Axis numbers display protein ladder (kDa). Flow cytometry experiments were performed in triplicate with each dot representing a technical replicate. Bars represent mean and error bars are ± standard deviation. IBs and IPs are representative of two independent experiments.

To examine the expression and function of each MR1 allomorph summarized in Table S1 and Figure 1, *A* and *B*, we stably transduced each *MR1* allele into endogenous MR1-deficient (MR1^null^) C1R and 293T cells (Fig. S1, *A*–*D*) (40, 41) and matched the resultant cell lines for comparable MR1 gene expression (Figs. 1, *C*–*E* and S1, *E*–*H*). MR1∗04 allelic variants containing MR1^R9H^ have been reported as having the single MR1^R9H^ mutation (18) or MR1^R9H^ combined with an additional MR1^H17R^ mutation (35). Here, we define the single (MR1^R9H^) allele as MR1∗04:01 and double allele (MR1^R9H/H71R^) as MR1∗04:02 using the principles of HLA nomenclature (42).

To assess the constitutive expression of MR1, we performed surface and intracellular staining of the C1R allomorph-expressing cell lines using the anti-MR1 monoclonal antibody (mAb) 26.5 (43). Transduced cells overexpressing MR1∗01, MR1∗02, MR1∗03 and MR1∗06 exhibited comparable surface and intracellular MR1 expression (Fig. 1, *D–F*). MR1∗04:01 and MR1∗04:02 had substantially greater total MR1 expressed with an approximate 2-fold increase in surface expression when compared MR1∗01 (Fig. 1, *D–F*). We previously demonstrated that MR1∗04 allomorphs, bearing the Arg9His mutation, preferentially presented pyridoxal (21). Thus, we hypothesized that the increase in MR1∗04:01 and MR1∗04:02 expression may be attributed to the supplementation of vitamin B6 within the cell culture media. Supporting this hypothesis, in media without vitamin B6 supplementation other than that present in fetal calf serum, we observed a 50% reduction in MR1 surface expression of the MR1∗04 allomorphs while only a 17% reduction with the wild type MR1∗01 allomorph, also known to bind B6 vitamers (21, 44) but at a lower level than MR1∗04 allomorphs (Fig. 1*G*) (21). This indicates that the supplementation of the media with vitamin B6 contributed to the elevated MR1∗04 expression in our cell line system as compared to other allomorphs.

The expression level of MR1∗05 on the surface (Fig. 1, *D* and *E*) and intracellularly (Fig. 1*F*) detected by 26.5 mAb was greatly reduced compared to the other allomorphs, indicating that the MR1∗05 polymorphisms considerably diminished the presentation of endogenous ligand(s). To determine if the observed reduction in the expression level of intracellular MR1∗05 resulted from misfolded protein undetectable by the 26.5 mAb, we performed an immunoblot using an antibody targeting the cytosolic tail (CT) of MR1. This antibody retains specificity for MR1 in a partially folded state within the endoplasmic reticulum (ER) making it suitable for detecting MR1∗05 (Fig. 1*H*) (14, 45). Using this approach, we observed that the levels of MR1∗05 in this cell line were similar to the expression detected in the MR1∗01 and MR1∗02 cell lines. This was inconsistent with our surface and intracellular staining data, supporting the hypothesis that MR1∗05 may be in a misfolded state (Fig. 1*I*). MR1∗03 and MR1∗06 both showed slightly greater expression of MR1 when compared to MR1∗01 (Fig. 1*I*). Consistent with surface and intracellular expression measured by flow cytometry, the amount of MR1 detected by immunoblots in cells transduced with either of the two MR1∗04 alleles was greater than other MR1 allomorphs (Fig. 1*I*).

To further validate that MR1∗05 was present within the cell in a misfolded or partially folded state, we performed immunoprecipitations using either the conformationally-sensitive 8F2.F9 (46) (Fig. 1*J*) or anti-MR1-CT (14) (Fig. 1*K*) mAbs followed by probing of these immune complexes by immunoblotting with anti-MR1-CT (Fig. 1, *J* and *K*). The MR1∗05 allomorph captured by MR1-CT immunoprecipitation was observed in the anti-MR1-CT immunoblot. However, the MR1∗05 allomorph was essentially undetectable in the 8F2.F9 immunoprecipitates following immunoblot with anti-MR1-CT (Fig. 1*K*). Together, these data suggest that there is a reduced amount of properly folded MR1∗05 in the steady state, and the majority of MR1∗05 present within the cell is in an incorrectly folded state that may be targeted for destruction in the ER, leading to the reduction in surface expression.

### Allomorphs of MR1 differ in their ligand-binding capacity

**Figure 2:**
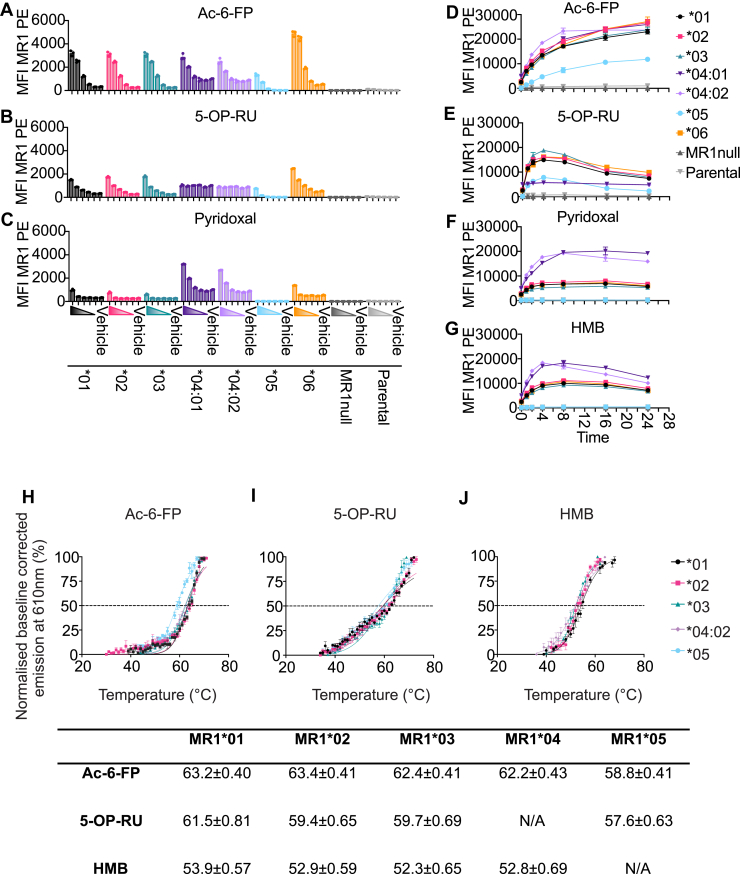
**Differential MR1 allomorph upregulation and stability in the presence of diverse ligands.***A–C*, surface expression of MR1 allomorph cell lines after overnight incubation with Ac-6-FP (*A*) or 5-OP-RU (*B*) titrated 10-fold from 10 μM, or pyridoxal (*C*) titrated 10-fold from 100 μM. Colour-coded wedges correspond to ligand titration from top dose to zero. Vehicle represents a DMSO control. Data are representative of two independent experiments performed in triplicate. Bars represent mean and error bars are ± standard deviation. *D–G*, line graphs representing surface expression of MR1 allomorph cell lines after incubation with 10 μM Ac-6-FP (*D*), 5-OP-RU (*E*) or 100 μM pyridoxal (*F*), HMB (*G*) at timepoints over 24 h. Data are representative of two independent experiments performed in triplicate. Symbols represent mean and error bars are ± standard deviation. *H–J*, thermal shift assay of different soluble MR1∗01–MR1∗05 proteins bound to Ac-6-FP (*H*), 5-OP-RU (*I*), and HMB (*J*). The mean Tm50 values are from three independent experiments performed in triplicate. Dots represent mean and error bars are ± standard deviation. Table summarizes the mean Tm50 values ± standard deviation.

To determine the ability of the MR1 allomorphs to egress from the endoplasmic reticulum (ER) to the cell surface in response to ligand, we incubated the MR1 allomorph overexpressing cell lines for 16 h with titrating doses of Ac-6-FP, 5-OP-RU or pyridoxal in standard culture media that includes B6 supplementation. The MR1∗01, MR1∗02, MR1∗03 and MR1∗06 expressing cell lines showed comparable MR1 upregulation in response to all three ligands (Fig. 2, *A*–*C*), revealing a hierarchy of ligand-dependent MR1 upregulation, with Ac-6-FP > 5-OP-RU >> pyridoxal as reported for the most common MR1∗01 (1, 21, 47). Notably, upregulation of MR1∗06 was greater than MR1∗01, particularly in the presence of Ac-6-FP (Fig. 2*A*). As reported previously, MR1 bearing the Arg9His mutation (shown here as MR1∗04:01 and MR1∗04:02) was not upregulated in response to 5-OP-RU (18), but was strongly upregulated in response to pyridoxal when compared with MR1∗01 (21) (Fig. 2, *B* and *C*) confirming the preferential binding of pyridoxal by the MR1∗04 allomorphs. The addition of Ac-6-FP or 5-OP-RU but not pyridoxal to the MR1∗05 overexpressing cell line induced only modest surface expression of MR1 (Fig. 2, *A–C*).

To evaluate the kinetics of the cell surface upregulation of the different MR1 allomorphs, we performed a 24-h time course using a single dose of Ac-6-FP, 5-OP-RU, pyridoxal or 2-hydroxy-5-methoxybenzaldehyde (HMB), a weak MR1∗01 drug-like ligand (19). MR1∗01, MR1∗02, MR1∗03, MR1∗05, and MR1∗06 allomorphs showed similar upregulation kinetics, with variable MR1 surface intensity for Ac-6-FP and 5-OP-RU (Fig. 2, *D* and *E*), consistent with previous findings for MR1∗01 expressing C1R cells (47, 48). In response to pyridoxal or HMB, surface expression of all MR1 allomorphs except MR1∗05 was greatest at 4 to 8 h, and then gradually reduced by 24 h (Fig. 2, *F* and *G*). MR1∗04 cell lines followed similar upregulation kinetics in response to Ac-6-FP (Fig. 2*D*) but as expected showed no detectable response to 5-OP-RU (Fig. 2*E*) (18). In response to pyridoxal or HMB, MR1∗04:01 and MR1∗04:02 cell lines showed the greatest upregulation with a peak surface expression at 8 h (Fig. 2*F*). Despite its low level of constitutive expression (Fig. 1, *D–F*), the MR1∗05 allomorph exhibited a modest upregulation in response to Ac-6-FP and 5-OP-RU, following a similar kinetic pattern, but with a lower magnitude than that was observed for the MR1∗01 allomorph (Fig. 2, *D* and *E*). However, in response to pyridoxal and HMB, there was no detectable increase in MR1 surface expression from MR1∗05 (Fig. 2, *F* and *G*). Collectively, these data indicate that ligands that potently upregulate MR1∗01 can partially rescue MR1∗05 surface expression, but less potent MR1∗01 ligands are insufficient to induce MR1∗05 upregulation, suggesting intrinsic instability or ligand-binding constraints of this allomorph. Therefore, we next examined the stability of soluble forms of each allomorph with known MR1 ligands.

### Allomorphs of MR1 have similar MR1–antigen complex stability

The extracellular portion of MR1∗06 is identical to that of MR1∗01 so MR1∗06 was not included in the thermostability assay. The stability of refolded recombinant protein from the MR1 allomorphs (MR1∗01–MR1∗05) was examined in solution in the presence of known high-affinity ligands for MR1∗01, including Ac-6-FP, 5-OP-RU, and the lower affinity ligand HMB. The protein stability of the various MR1-ligand complexes was determined using a thermal shift assay (48). All MR1 allomorphs from MR1∗01 to MR1∗05 were successfully refolded in the presence of Ac-6-FP, forming the most stable complexes with melting temperatures (Tm) ranging from 58.8 to 63.4 °C (Fig. 2*H*). Similarly, all of the allomorphs, with the exception of MR1∗04, were refolded with 5-OP-RU and displayed Tm values comparable to that of MR1∗01-5-OP-RU (61.5 °C) (Fig. 2*I*). These findings indicate that the MR1 polymorphisms do not compromise the stability of MR1-ligand complexes with strong binders. In contrast, the purified MR1-HMB complexes showed reduced Tm values (∼52.3–53.9 °C) reflecting the weaker affinity nature of this MR1 ligand (Fig. 2*J*). Unlike the other MR1 allomorphs, MR1∗05 did not refold in the presence of HMB (Fig. S2*A*) but did refold in the presence of 5-OP-RU and Ac-6-FP (Fig. S2, *B* and *C*). Together, these data suggest that while most MR1 polymorphisms do not impact MR1 stability, the MR1∗05 polymorphisms may restrict the ligand repertoire, favoring strong MR1 binders and failing to stabilize in the presence of weaker MR1 ligands.

### Structural analysis of MR1 allomorphs reveals a key interaction is ablated in the MR1∗05 allomorph

**Figure 3:**
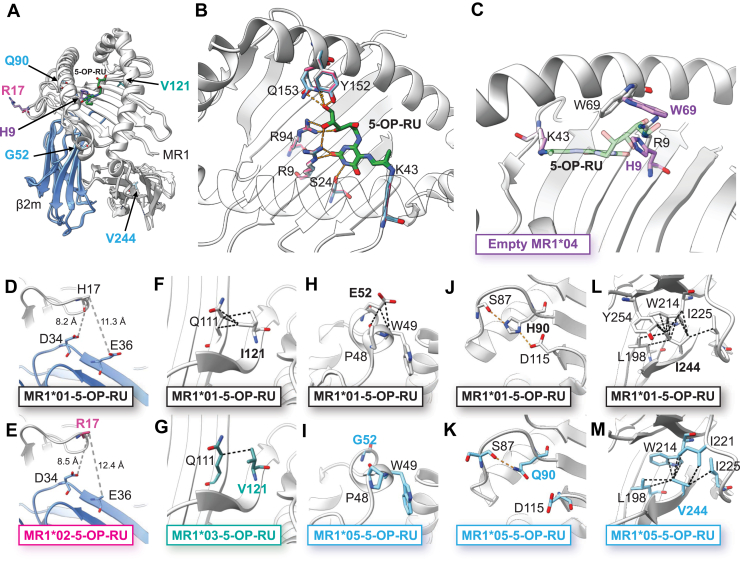
**Crystal structures of allelic variants in MR1 identify critical residue interactions, not present in MR1∗01.***A*, superposition of MR1∗02-5-OP-RU, MR1∗03-5-OP-RU, empty MR1∗04, MR1∗05-5-OP-RU and MR1∗01-5-OP-RU (PDB:6PUC). MR1 heavy chain for each allomorph is depicted in *light grey*, β2m in *blue* and polymorphic MR1 residues are shown as sticks. *B*, close-up of the antigen-binding cleft of MR1∗02, MR1∗03 and MR1∗05 bound to 5-OP-RU superimposed onto that of MR1∗01. *C*, superposition of the binding cleft of empty MR1∗04 and MR1∗01-5-OP-RU illustrating the Arg9His polymorphism, inducing a conformational shift in Tyr69 occluding the binding of 5-OP-RU. *D–M*, the detailed structures of various residues in MR1∗01 (*upper panels*) and the MR1 allomorphs (*lower panels*). *D* and *E*, the His17 and Arg17 residues in MR1∗01 (*D*) and MR1∗02 (*E*) appear to be very dynamic and part of a flexible loop region in the α1 domain. The His17/Arg17 do not impact the MR1-β2m interface noting the distance between β2m Asp34 and Glu36. *F* and *G*, localized effect of the Ile121Val polymorphism harbored by MR1∗03 on the underside of the MR1 binding groove results in the reduction of hydrophobic contacts (*black dotted lines*) with MR1 Glu111. *H* and *I*, MR1∗05 carries a Glu52Gly substitution in the α1 helix. The loss of the Glu52 side chain in MR1∗05 disrupts hydrophobic contacts with Pro48 and Trp49. *J* and *K*, additionally, MR1∗05 carries a His90Gln polymorphism in the α2 helix, disrupting a hydrogen bond with Asp115 and causing a conformational shift in this aspartic acid. The Gln90 in MR1∗05 can still form a hydrogen bond with Ser87, as illustrated by the orange dashed line. *L* and *M*, the MR1∗05 Ile244Val mutation in the α3 domain induces the loss of hydrophobic contacts between Trp214 and Tyr254.

To explore the impact of MR1 polymorphisms on the interactions within the MR1 antigen-binding cleft and at the interface with the MAIT TCR, we determined crystal structures of MR1∗02-5-OP-RU, MR1∗03-5-OP-RU, MR1∗05-5-OP-RU and unloaded MR1∗04 in complex with MAIT A-F7 TCR (Fig. 3 and Table S2). Despite the presence of different MR1 polymorphisms, structural comparison of these soluble MR1 variants revealed a high degree of conservation in the overall MR1-β2m fold and TCR docking that was well-preserved across the different MR1 allomorphs.

For all liganded structures, there was clear electron density permitting the unambiguous modelling of 5-OP-RU within the MR1 binding pocket, where a Schiff base bond with Lys43 was clearly observed (Fig. 3*A*). For MR1∗02, MR1∗03 and MR1∗05 structures, 5-OP-RU was effectively enclosed by conserved network of hydrophobic and hydrogen-bond interactions involving MR1 residues Ser24, Arg9, Arg94, Glu153 and Tyr152, in a manner akin to the MR1∗01-5-OP-RU structure (48) (Fig. 3*B*). Additionally, we determined the structure of unliganded MR1∗04:01, containing the Arg9His mutation within the antigen binding cleft. This mutation induces a conformational shift of the side chain of Trp69 that sterically clashes with 5-OP-RU inhibiting its binding to either MR1∗04:01 or MR1∗04:02 (Fig. 3*C*), as observed in our previously published MR1^R9H^ structure (18).

Although minimal differences were observed in the global MR1 architecture, localised effects at these polymorphic sites may subtly affect protein stability. MR1∗02 contains a His17Arg mutation located within a surface-exposed and flexible loop region adjacent to the MR1-β2m interface (Fig. 3, *D* and *E*). In the MR1∗02 structure, there was weak density surrounding Arg17, and similarly, the side chain of His17 in the MR1∗01 structure is poorly resolved, indicating intrinsic flexibility at this position. The His17Arg mutation is unlikely to impact MR1-β2m association or the stability of the heterodimer interface, consistent with the notable lack of conservation across different species and the thermostability measurements shown in Figure 2, *H*–*J*. In contrast, MR1∗03 features an Ile121Val mutation on the periphery of the antigen-binding groove (Fig. 3, *F* and *G*). The smaller valine side chain disrupts non-polar interactions with Gln111 and leads to some loss of hydrophobic contacts (Fig. 3*G*). Regardless of the net decrease in hydrophobic packing, Val121 retains proximity to Gln111 and does not induce any conformational changes to the antigen-binding cleft.

The *MR1∗05* allele encodes polymorphisms across the α1 (Glu52Gly), α2 (His90Gln) and α3 (Ile244Val) domains of MR1. In the MR1∗01 structure, Glu52 contributes to local packing in the α1 domain primarily through van der Waals interactions with Pro48 and Trp49 (Fig. 3*H*). Consequently, the Glu52Gly polymorphism disrupts these packing interactions (Fig. 3*I*). Additionally, proximal to the F′ pocket in the α2 domain, MR1∗05 contains a His90Gln polymorphism that induces a conformational shift in the neighboring Asp115 abolishing the hydrogen bond observed between His90 and this aspartic acid residue found in MR1∗01 (Fig. 3, *J* and *K*). This hydrogen bond between His90 and Asp115 in the MR1∗01 structure is likely stabilized by an electrostatic attraction (Fig. 3*J*), whereas the substitution with a glutamine eliminates this interaction (Fig. 3*K*). The loss of this stabilising interaction near the MR1 F′ pocket may explain the reduced MR1∗05 expression observed in the cell lines and Fig. S1, *B* and *D* and *F*). In MR1∗01, Ile244 is buried within the hydrophobic β-sheet of the α3 domain, forming multiple van der Waal interactions with neighboring hydrophobic residues, including Leu198, Trp214, Ile221, Ile225, and Tyr254 (Fig. 3*L*). The smaller side chain of valine slightly reduces its hydrophobic contacts within the α3 domain (Fig. 3*M*). Despite this decrease in non-polar interactions, the Ile244Val mutation is not likely to impact protein stability nor affect MR1-β2m interactions. Interestingly, the presence of Val244 in mouse, rat, pig, bovine and sheep MR1 (Fig. S3) (4, 5, 6) may suggest some evolutionary variability of this residue.

Overall, our structural data reveal that these MR1 polymorphisms do not impact MR1 architecture. Rather, these substitutions, particularly the His90Gln substitution in MR1∗05, may have localized effects on MR1 stability as reflected in the impaired cell surface expression and ligand-induced upregulation and, to a lesser extent, in the thermal stability assays (Fig. 2).

### Instability of MR1∗05 allomorph is controlled by the His90Gln and Glu52Gly substitutions

**Figure 4:**
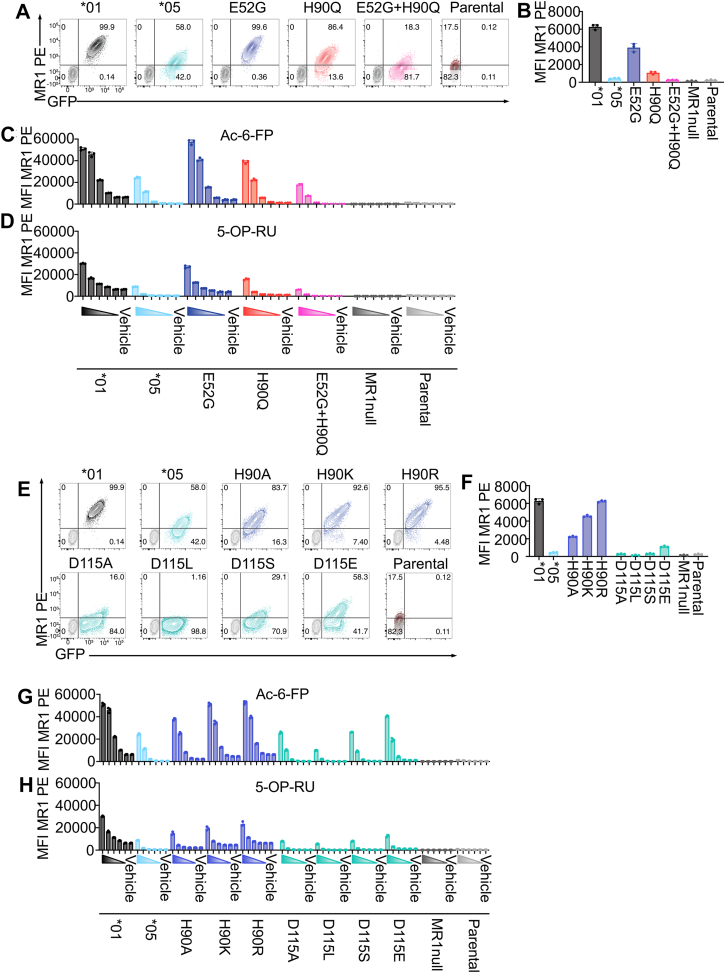
**MR1∗05 mutations Glu52Gly and His90Gln synergistically control MR1∗05 expression.***A*, representative flow cytometry panels showing C1R.MR1^null^ cell lines transduced with MR1∗01 (*black*), MR1∗05 (*blue*), MR1 Glu52Gly (*dark blue*), MR1 His90Gln (*red*) and MR1 Glu52Gly + His90Gln (*pink*) and C1R parental cells (*dark red*) compared to with C1R.MR1^null^ cell line (*grey*) displaying median fluorescence intensity (MFI) of anti-MR1-PE surface staining (*y axis*) and GFP (*x axis*). *B*, histogram displaying MR1 surface expression of each cell line. *C* and *D*, histograms representing MR1 surface expression from each mutant cell line incubated for 16 h with Ac-6-FP (*C*) or 5-OP-RU (*D*) titrated 10-fold from 10 μM or DMSO (vehicle). Colour-coded wedges correspond to ligand titration from top dose to zero. *E*, representative flow cytometry panels showing surface staining with anti-MR1-PE (*y axis*) and GFP (*x axis)* for C1R.MR1^null^ cell lines transduced with MR1∗01 (*black*), MR1∗05 (*blue*), MR1∗01-His90Ala, -His90Lys, -His90Arg (*dark blue*), MR1∗01-Asp115Ala, -Asp115Leu, -Asp115Ser, -Asp115Glu (*green*) and C1R parental cells (*dark red*) compared to the C1R.MR1^null^ cell line (*grey*). *F*, Histogram representing MR1 surface expression of each mutant cell line. *G* and *H*, histograms representing MR1 surface expression from each mutant cell line incubated for 16 h as described above. Data are derived from two experiments performed in triplicate, with each dot representing a technical replicate. C1R.MR1^null^, C1R parental, MR1∗01 and MR1∗05 control cell line datasets are reused in panels *A–D* and panels *E–H*. Bars represent the mean, and error bars are ± standard deviation.

As the non-conservative Glu52Gly and His90Gln polymorphisms in MR1 are the most likely candidates responsible for the MR1∗05 phenotype, we generated cell lines overexpressing MR1 with Glu52Gly and His90Gln single and double mutations (Fig. 4*A*). We observed that the constitutive MR1 expression was reduced by 38% for the single MR1-Glu52Gly mutant and 83% for the single MR1-His90Gln mutant when compared to MR1∗01 (Fig. 4*B*). In comparison, the MR1-Glu52Gly/His90Gln double mutant reproduced the severe loss of surface MR1 observed for the MR1∗05 cell line (Fig. 4, *A* and *B*), indicating that both mutations contributed to the MR1∗05 phenotype, with His90Gln having the greatest effect. We next examined the response of the single and double MR1∗05 mutants to ligand-dependent upregulation, using Ac-6-FP and 5-OP-RU (Fig. 4, *C* and *D*). The dose-dependent MR1-Glu52Gly response to ligands induced surface expression resembling MR1∗01, while MR1-His90Gln and MR1-Glu52Gly/His90Gln mutants were more comparable to MR1∗05 (Fig. 4, *C* and *D*). Together, these data confirm that altered surface expression of MR1∗05 results primarily from His90Gln and, to a lesser degree, Glu52Gly polymorphisms spanning the MR1 α1/2-domains.

To investigate the importance of His90 interactions, we generated mutant MR1∗01 overexpressing cell lines with single amino acid changes targeting either His90 or Asp115, which formed an electrostatic interaction that was ablated in MR1∗05 (Fig. 3, *J* and *K*). We mutated His90 and Asp115 with simple (His90Ala and Asp115Ala), charged (His90Lys, His90Arg and Asp115Glu), or uncharged (Asp115Ser and Asp115Leu) side chains (Fig. 4*E*). Interestingly, MR1-His90Arg did not appreciably alter MR1 surface expression, suggesting the Arg90 sidechain may maintain the interaction with Asp115. However, cell lines expressing the mutations MR1-His90Lys and MR1-His90Ala both had modestly reduced levels of MR1 surface expression compared to MR1∗01, though not as low as MR1∗05 (Fig. 4, *E* and *F*). In contrast, all cell lines expressing MR1-Asp115 substitutions showed a substantial loss of MR1 surface expression, which closely resembled the MR1∗05 overexpressing cell line (Fig. 4, *E* and *F*). We observed that the mutations targeting MR1-His90 followed a similar upregulation pattern to MR1∗01, but with a slight reduction in surface expression, consistent with the reduced constitutive surface expression (Fig. 4, *E* and *F*). We next examined the capacity of MR1 ligands 5-OP-RU and Ac-6-FP to restore MR1 cell surface expression (Fig. 4, *G* and *H*). All cell lines expressing MR1-Asp115 mutants showed poor upregulation of MR1 in response to the titrating ligands in a pattern largely consistent with MR1∗05 (Fig. 4, *G* and *H*). Together with the impaired expression of the MR1-His90Gln single mutant (Fig. 4, *A*–*D*), these data provide evidence that the interaction between Asp115 and His90 is likely crucial for MR1 stability and cell surface expression in the absence of strong binding ligands.

### MR1 allomorphs have similar affinities and conserved interactions with MAIT TCRs

**Figure 5:**
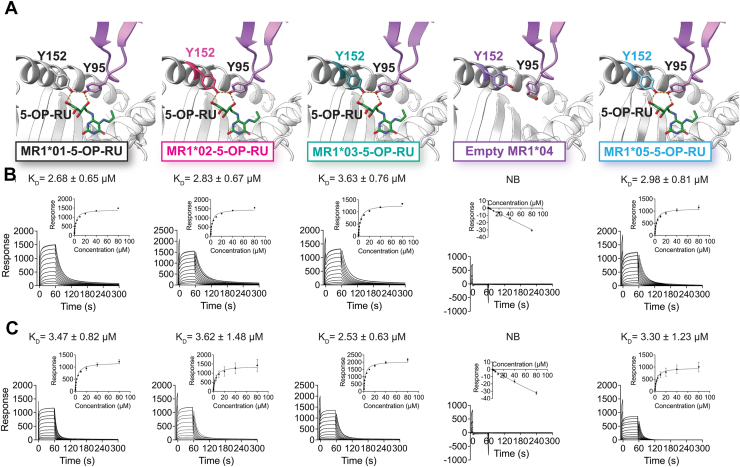
**MR1-MAIT TCR affinities and interactions are comparable across different allelic variants of MR1.***A*, structures of MAIT TCR Tyr95α interactions with MR1∗01-5-OP-RU, MR1∗02-5-OP-RU, MR1∗03-5-OP-RU, empty MR1∗04 and MR1∗05-5-OP-RU. The interaction triad between TCRα Tyr95, MR1 Tyr152, and 5-OP-RU is highly conserved and unaffected by the MR1∗02, MR1∗03 and MR1∗05 polymorphisms, whilst there is no interaction between the unliganded MR1∗04 and A-F7. Steady-state affinity measurements of MAIT TCRs A-F7 (*B*) and BV6-4 (*C*) against different MR1 allomorphs including MR1∗01-5-OP-RU, MR1∗02-5-OP-RU, MR1∗03-5-OP-RU, unliganded MR1∗04 and MR1∗05-5-OP-RU. The SPR sensograms, equilibrium curves and the equilibrium dissociation constants values (K_D_) were prepared in GraphPad Prism, assuming 1:1 binding. All titrations were performed in duplicate for two independent experiments.

We next examined whether the MR1 polymorphisms influenced binding of the MAIT TCR. In the crystal structures of the MAIT A-F7 TCR complexed with the MR1 allomorphs, we observed no differences in MAIT TCR docking when compared to MR1∗01. Further, the critical interaction triad (48) formed between 5-OP-RU, Tyr152 of MR1 and the Tyr95 of TCRα chain remained intact across all ligand binding allomorphs (Fig. 5*A*). Next, we assessed the binding of the MR1 allomorphs refolded with 5-OP-RU to MAIT TCRs, A-F7 and BV6-4, using surface plasmon resonance (SPR) (Fig. 5, *B* and *C*). The MR1∗02, MR1∗03 and MR1∗05 allomorphs bound to 5-OP-RU had comparable affinities for the A-F7 TCR compared with MR1∗01-5-OP-RU (2.7–3.6 μM) (Fig. 5*B*). Similarly, there was no significant difference in the dissociation constant (K_D_) values across the MR1∗01, MR1∗02, MR1∗03 and MR1∗05 complex binding to BV6-4 with K_D_ ranging from 2.5 to 3.6 μM (Fig. 5*C*). Furthermore, empty MR1∗04 showed no binding to either of these TCRs (Fig. 5, *B* and *C*). Taken together, our structural and binding analyses reveal that the MR1 polymorphisms, apart from MR1∗04, do not impair MAIT TCR recognition of 5-OP-RU, consistent with the observation that none of the polymorphic residues map to the TCR docking site.

### Allomorphs of MR1 differentially activate MAIT and diverse MR1-reactive T cells

**Figure 6:**
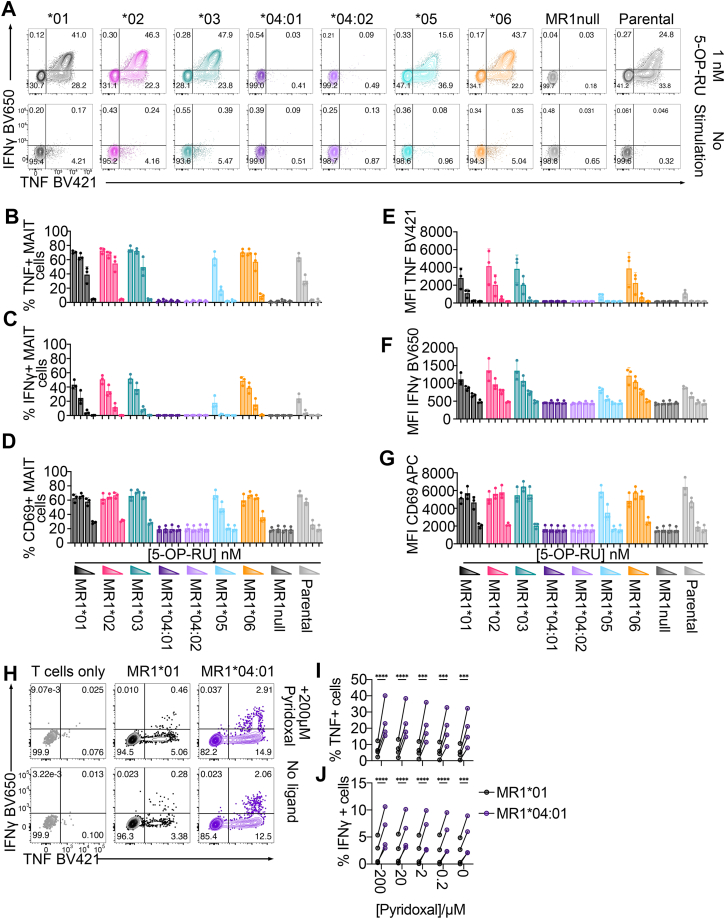
**Differential activation of T cells by diverse ligands presented by MR1 allomorphs.***A*, representative flow cytometry panels showing MAIT cell intracellular staining with anti-IFNγ (*y axis*) or anti-TNF (*x axis*) antibodies after stimulation with MR1 allomorph C1R cell lines, C1R.MR1^null^ cells or C1R parental cells in the presence or absence of 1 nM 5-OP-RU. *B–D*, histograms display the percentage of TNF (*B*), IFNγ (*C*) or CD69 (*D*) expression among MAIT cells (TRAV1-2+CD161+) incubated with MR1 allomorph cell lines pulsed with 5-OP-RU titrated 10-fold from 1 nM or in the absence of ligand. Colour-coded wedges correspond to 5-OP-RU titration from top dose to zero. *E–G*, histograms display the intensity (MFI) of TNF (*E*), IFNγ (*F*), or CD69 (*G*) from MAIT cells as above. Data are from one experiment with three healthy blood donors. Dots represent individual donors; bars represent mean and error bars are ± standard deviation. *H*, representative flow cytometry panels showing intracellular staining with anti-IFNγ (*y axis*) or anti-TNF (*x axis*) from expanded MR1^R9H^-pyridoxal tetramer-reactive T cells cultured alone (*grey*) or after stimulation with C1R.MR1^null^ cell lines transduced with MR1∗01 (*black*), MR1∗04:01 (*dark purple*) pulsed with 200 μM pyridoxal (top panels) or in the absence of exogenous ligand (*bottom panels*). *I* and *J*, paired analyses display the percentage of TNF (*I*) or IFNγ (*J*) expressing T cells after stimulation as above. Data are from four separate healthy blood donors. Dots represent individual donors; bars represent mean and error bars are ± standard deviation. Statistical significance was determined using a two-way ANOVA with Sidak’s multiple comparison test.

Considering that MR1∗05 surface expression could be restored in the presence of 5-OP-RU (Figs. 2 and 4), and that the canonical MAIT TCRs A-F7 and BV6-4 recognised the MR1∗05-5-OP-RU complex similarly as MR1∗01-5-OP-RU (Fig. 5, *A*–*C*), we next examined the capacity for the MR1 allomorphs to activate primary MAIT cells. We isolated MAIT cells from healthy donor peripheral blood mononuclear cells (PBMCs) *via* magnetic enrichment of TRAV1-2+ cells (Fig. S4, *A*–*C*). TRAV1-2+ enriched cells were then cultured with the MR1 allomorph cell lines pulsed with titrating doses of 5-OP-RU. Production of TNF and IFNγ cytokines, and the expression of the early activation receptor CD69 were examined. From three donors, we observed a comparable percentage of MAIT cell activation by all readouts in response to cell lines expressing MR1∗01, MR1∗02, MR1∗03 and MR1∗06 incubated with titrating doses of 5-OP-RU (Fig. 6, *A*–*D*). In addition, the intensity (MFI) of CD69 positive and cytokine-producing MAIT cells was similar in response to these cell lines across each of the titrating Ag doses (Fig. 6, *E*–*G*). These data indicate that the mutations present in the MR1∗02, MR1∗03 and MR1∗06 allomorph cell lines do not impact MAIT cell activation. At the highest dose of 5-OP-RU, MR1∗05 induced a similar percentage of MAIT cells expressing TNF (Fig. 6*B*) and CD69 (Fig. 6*D*), but not IFNγ (Fig. 6*C*), when compared to MR1∗01. At all other doses of 5-OP-RU, the percentage of cytokine-producing and CD69-positive MAIT cells was considerably reduced (Fig. 6, *B*–*D*). Among activated MAIT cells (Fig. 6, *E*–*G*), there was a marked reduction in the amount of cytokine produced and surface CD69 expressed (MFI) with MR1∗05, indicating that the potency of this response was attenuated compared to MR1∗01.

We previously showed that CD8 is an important coreceptor during MR1-MAIT TCR engagement that enhances the intensity of MAIT cell responses (40). To determine if MR1 polymorphisms could impact CD8 engagement, we stratified MAIT cells from each PBMC donor based on coreceptor expression (Fig. S4, *D*–*F*) and examined the cytokine responses of CD8-positive and CD4/CD8 double-negative (DN) MAIT cells. MR1∗01, MR1∗02, MR1∗03 and MR1∗06 showed no significant differences in the percentage of TNF and IFNγ-producing MAIT cells between the CD8-positive and DN cell subsets. However, MR1∗05-stimulated MAIT cells showed a significant increase in the percentage of TNF and IFNγ-positive cells at the top dose of 5-OP-RU in CD8-positive MAIT cells compared to DN MAIT cells (Fig. S4, *E* and *F*). These data indicate that the MAIT cell response to MR1∗05 is more sensitive to the presence of CD8, inferring that CD8 engagement of MR1∗05 is preserved.

Consistent with our previous findings (18), MAIT cells were not activated by MR1 cell lines expressing the Arg9His mutation (MR1∗04:01 and MR1∗04:02) (Figs. 6, *A*–*G* and S4, *E* and *F*). However, MR1∗04 can still present ligands to other T cells (21, 49). To examine the antigen presentation capacity of MR1∗04 to primary T cells, we produced MR1^R9H^-tetramers loaded with pyridoxal and enriched tetramer-positive cells from the PBMCs of an additional four healthy donors. The majority of enriched T cells from all donors were found to express the CD8 coreceptor (>80%; Fig. S4, *G* and *H*). Due to the low frequency of non-MAIT MR1-reactive T cells in human blood (50, 51, 52), we sorted MR1^R9H^-pyridoxal tetramer-positive T cells prior to *in vitro* expansion (Fig. S4*I*). Expanded MR1-reactive T cells were predominantly TRAV1-2 negative, and >90% of the expanded T cells in all donors lacked the surface marker CD161 expressed by MAIT cells (Fig. S4, *J* and *K*). As tetramer binding does not always correlate with T cell function (40), we stimulated the expanded T cells with MR1∗01 or MR1∗04:01 overexpressing cell lines in the presence or absence of titrating doses of pyridoxal and measured the production of TNF and IFNγ cytokines (Fig. 6, *H*–*J*). In all four donors, a greater proportion of the expanded T cells produced TNF (Fig. 6, *H* and *I*) and IFNγ (Fig. 6, *H* and *J*) in response to MR1∗04:01 compared to MR1∗01, revealing a clear preference for MR1∗04:01 by these T cells. Notably, the further addition of pyridoxal to the culture induced only a modest increase in T cell activation (Fig. 6, *H*–*J*). Together, these data demonstrate that the MR1∗04:01 allomorph is functional, with the capacity to present B6-related antigens and potentially other self-metabolites that stimulate T cells.

### Infection with HSV differentially impacts MR1 expression of MR1 allomorphs

**Figure 7:**
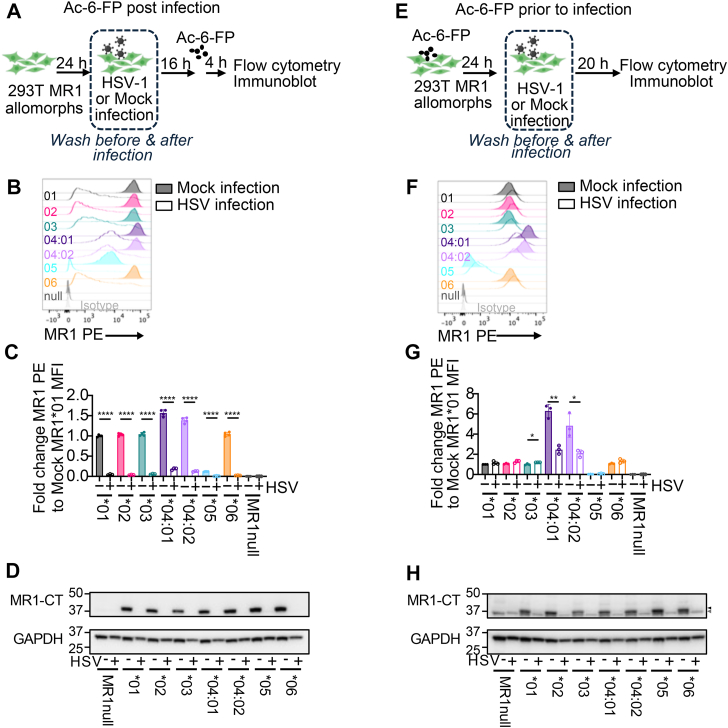
**Infection with HSV differentially impacts MR1 surface expression.***A*, schematic relating to (*B–D*) outlining infection of 293T.MR1^null^ cell lines expressing each MR1 allomorph with HSV-1 strain F, at a multiplicity of infection (MOI) of 2. After 1 h of virus adsorption the cells were washed and the media replaced. Cells were treated with 5 μM Ac-6-FP 4 h prior to harvesting for flow cytometry (*B* and *C*) or immunoblot (*D*) at 20 h post infection. *B*, representative flow cytometry histograms showing MR1 surface expression following mock (shaded) or HSV-1 infection (unshaded) of cell lines transduced with MR1∗01 (*black*), MR1∗02 (*pink*), MR1∗03 (*green*), MR1∗04:01 (*dark purple*), MR1∗04:02 (*light purple*), MR1∗05 (*blue*), MR1∗06 (*orange*) and 293T.MR1^null^ cell line (*grey*). *C*, fold change of the MFI of anti-MR1-PE of each allomorph cell line compared to mock-infected 293T.MR1^null^ MR1∗01 cells. *D*, immunoblots (IB) from lysed MR1 allomorph cell lines post HSV or mock infection with detection of MR1 using anti-MR1-CT or control GAPDH. Axis numbers display protein ladder (kDa). *E*, schematic relating to (*F–H*) outlining 293T.MR1^null^ cell lines pre-treated with 5 μM Ac-6-FP for 24 h, washed and infected HSV-1 strain F at a MOI of 2. After 1 h of virus adsorption the cells were washed and the media replaced. Cells were harvested for flow cytometry (*F* and *G*) or IB (*H*) at 20 h post infection. *F*, representative flow cytometry histograms showing MR1 surface expression following mock (shaded) or HSV-1 infection (unshaded) of 293T.MR1^null^ cell lines as outlined in (*E*). *G*, Fold change of the MFI of anti-MR1-PE of each allomorph cell line compared to mock-infected 293T.MR1^null^ MR1∗01. *H*, IB from lysed MR1 allomorph cell lines post HSV or mock infection with detection of MR1 using anti-MR1-CT or control GAPDH. Axis numbers display protein ladder (kDa). Of the two bands present in the MR1-CT IB the lower molecular weight band (*grey triangle*) corresponds to remaining GAPDH signal from the prior probe whereas the higher band (*black triangle*) is the new MR1-CT probe. Flow cytometry experiments are representative of two independent experiments performed in triplicate with each dot representing an experimental replicate. Bars represent mean and error bars are ± standard deviation. Statistical significance was calculated by unpaired Student’s *t* test. ∗∗∗∗*p*< 0.0001, ∗∗*p*< 0.01, ∗*p*< 0.05. IBs are representative of two independent experiments.

We have previously demonstrated that human herpesviruses modulate MR1∗01 expression and antigen presentation, impacting the activation of MAIT cells (29, 30). HSV-1 encodes multiple genes that target different stages in the MR1∗01 antigen presentation pathway to rapidly inhibit surface MR1 antigen presentation (29, 53, 54). To evaluate the capacity of HSV-1 to modulate the different MR1 allomorphs, 293T cells overexpressing the MR1 allomorphs were infected with HSV-1, sufficient to infect close to 100% of the cells. To ascertain viral suppression of newly synthesized, ER-resident MR1 *versus* Ag-loaded pre-formed MR1, we carried out separate infections whereby the cells were loaded with Ag (Ac-6-FP) either post-infection, after full expression of the viral genome (Fig. 7, *A*–*D*), or prior to infection (Fig. 7, *E*–*H*). As previously shown for MR1∗01 (29), incubating the 293T transfectants with Ag for the final 4 h of the 20-h infection (Fig. 7*A*) does not protect nascent or Ag-loaded MR1 from virus suppression of MR1 surface expression (Fig. 7, *B* and *C*). Relative differences in the amount of surface MR1 allomorph expression in mock-infected 293T cells (Fig. 7, *B* and *C*) were comparable to those observed in the C1R MR1 allomorph cells treated with Ac-6-FP for the same amount of time (Fig. 2*D*). Specifically, the highest surface expression was associated with MR1∗04:01 and MR1∗04:02 expressing cell lines, while the lowest surface expression was MR1∗05, with comparable levels of surface MR1 demonstrated by the other allomorphs. Surface MR1 expression of all the allomorphs was significantly downregulated in the HSV-1-infected cells. Interestingly, the extent of downregulation of MR1∗04:01 and MR1∗04:02 was attenuated in comparison to the other allomorphs.

The loss of surface MR1 is primarily driven by rapid depletion of ER-resident MR1 that occurs within the first few hours of infection (29). Consequently, we evaluated total MR1 by immunoblot using the MR1-CT antibody at the same timepoint. Allomorph gene expression, as measured by raw GFP, was more consistent in the 293T cells than the C1R cells (Fig. S1, *A* and *C*). Correspondingly, MR1 levels in the mock-infected samples (Fig. 7*D*) were more consistent between the different 293T allomorphs than in the C1R cells (Fig. 1, *I* and *J*). MR1 was not detected in the HSV-1 infected lysates (Fig. 7*D*), confirming the capacity of the virus to effectively deplete intracellular reservoirs of all the MR1 allomorphs.

While HSV-1 effectively diminishes the level of immature MR1, the molecules that exit the ER prior to HSV-1 infection are protected from viral downregulation and exhibit moderately impaired endocytosis from the plasma membrane (53). By treating cells with Ac-6-FP only for the 24 h prior to infection (Fig. 7*E*), pre-existing surface MR1 can be maximized to reveal ligand-stabilized MR1 that is resistant to viral suppression (Fig. 7, *F* and *G*). Consistent with previous findings for MR1∗01 (29, 53) surface MR1∗01, MR1∗02, MR1∗03 and MR1∗06 was preserved or significantly upregulated (mock *versus* HSV infection, Fig. 7, *F* and *G*). Under these same conditions, MR1∗05 expression approached that of MR1^null^ in both the mock and infected cells, reflecting the combinatorial effect of its impaired steady-state cell surface expression and the removal of extracellular ligand at the time of infection. By contrast, the relative preservation of surface MR1∗04:01 and MR1∗04:02 was significantly reduced. Despite the detection of high surface MR1 in all except MR1∗05, total expression of MR1 in the HSV-1 infected cells was difficult to detect by immunoblot (MR1-CT) (Fig. 7*H*) due to the depletion of intracellular MR1.

Regardless of when Ac-6-FP was added to cells, surface MR1∗04:01 and MR1∗04:02 were expressed at substantially higher levels than MR1∗01, reflecting their enhanced capacity to bind the vitamin B6-related ligands present in the media throughout the assays. Collectively, these data demonstrate a partial preservation of cell surface expression of MR1∗04:01 and MR1∗04:02 molecules in the face of infection with HSV-1, while other MR1 allomorphs are profoundly susceptible to suppression of their expression by HSV-1.

## Discussion

In contrast to the highly polymorphic MHC class I and II antigen-presenting molecules (32), MR1 is generally described as being monomorphic and tailored toward presentation of a limited repertoire of riboflavin-derived metabolites to MAIT cells. Functionally, MAIT cells contribute to protection from microbial pathogens (55, 56, 57, 58, 59, 60, 61, 62, 63, 64, 65, 66), enhanced tissue repair (25, 67, 68, 69, 70), and modulation of T cell immunity in a variety of contexts, including graft *versus* host disease (71), autoimmunity (68), and tumor immunity (72, 73, 74, 75, 76, 77). The MR1 sequence is highly conserved between species and has co-evolved with TRAV-1, the MAIT TCRα variable gene (5, 6, 8), suggesting that MR1 has been maintained for presenting a limited number of microbial metabolites to MAIT cells. However, the number of defined MR1 ligands is growing and includes a diversity of ligands that are presented to T cells that may have roles outside antimicrobial immunity (19, 20, 21, 22, 23, 27, 50, 78). The physiological function of many of these ligands is unclear, but it is notable that natural alleles of MR1 have recently been described (18, 35, 79), provoking us to consider how newly defined MR1 polymorphisms may have functional relevance in relation to diverse ligand presentation to T cells.

Here we demonstrated that, albeit limited, polymorphisms in MR1 can indeed influence its function and ligand repertoire. However, of the seven alleles described to date, four of these (MR1∗01, MR1∗02, MR1∗03 and MR1∗06) are highly conserved in relation to their structure and functional antigen presentation capacity. Thus, MR1∗01, MR1∗02, MR1∗03 and MR1∗06 were all constitutively expressed on the cell surface at similar levels, presumably stabilized by endogenous or media-derived ligands. In other respects, MR1∗02, ∗03 and ∗06 were also functionally identical to the most common MR1∗01, in terms of intracellular MR1 expression, ligand-induced upregulation of cell surface MR1 and MAIT cell activation.

The study confirmed previous reports that MR1∗04 (comprising two alleles, MR1∗04:01 and MR1∗04:02) does not present 5-OP-RU to MAIT cells (18), but presents the alternate vitamin B6-derived ligands to T cells (21, 44). Specifically, we revealed that T cells from healthy donors isolated using pyridoxal-loaded MR1^R9H^ tetramers were predominantly non-MAIT MR1-reactive T cells and were preferentially activated in response to MR1∗04 *versus* MR1∗01. This difference in MR1 responsiveness may reflect preferential binding of MR1∗04 to pyridoxal and a tendency for some diverse MR1-reactive T cells to exhibit cross-reactivity to MR1∗04. Indeed, a T cell clone, MC.7.G5 (26), was expanded from primary PBMCs in response to stimulation by the A549 adenocarcinoma cell line, which is heterozygous for MR1∗01 and MR1∗04 (38). Later studies demonstrated that MC.7.G5 displayed enhanced activation in the context of MR1∗04 expressing APCs (21, 38). Taken together, our data provide further evidence that MR1∗04 is a functional antigen-presenting molecule, which accommodates a distinct antigen repertoire to that of MR1∗01. Nonetheless, this allele is rare in populations studied to date, and it remains unclear whether this has been evolutionarily selected for its immune function.

In contrast to MR1∗01, MR1∗02, MR1∗03, MR1∗04 and MR1∗06, the MR1∗05 allele confers a substantial loss of function controlled by two (Glu52Gly and His90Gln) of three mutations that distinguish MR1∗05 from the most prevalent MR1∗01. These mutations lead to a reduction in MR1 surface expression and impaired Ag presentation and T cell activation in the presence of 5-OP-RU. *In vitro* mutagenesis revealed that Gln90 was primarily responsible for these effects by disrupting an electrostatic bridge with Asp115 across the two MR1 α-domains. Both His90 and Asp115 are conserved in MR1 across all species analyzed, suggesting a critical role in the folding of MR1. Indeed, our data suggest that the loss of this interaction has implications for the folding and stability of MR1∗05 within the ER, impairing its antigen presentation capacity, particularly for ligands that induce modest MR1∗01 upregulation (19, 20, 21, 22, 24, 48, 80). The low expression of MR1∗05 on the cell surface and the absence of MR1∗05 in immunoprecipitates with conformationally-sensitive antibodies (46) suggest that MR1∗05 molecules are retained in the ER in a partially folded state (14). It is known that ligand neutralization of MR1-Lys43 by Schiff base within the MR1 binding pocket contributes to conformational changes that complete proper MR1 folding and association with β_2_m, thus triggering egress from the ER to the cell surface (14). In addition, other ligand interactions within the MR1 binding pocket also contribute to this stability and further assist in chaperone protein binding and trafficking to the cell surface (45, 81). One or more of these interactions is likely to be disrupted in MR1∗05.

While we observed diminished MAIT cell activation by MR1∗05 compared to MR1∗01, this response was amplified by MR1 overexpression in our cell line system. We predict MR1∗05 may resemble a near null allele at physiological expression that would likely have significant implications for MAIT and other MR1-restricted T cell development in homozygous individuals. Conversely, we hypothesize that expression of one copy of MR1∗01, or the functionally conserved MR1∗02, MR1∗03, MR1∗06 alleles, would be sufficient to compensate for the reduced functionality of MR1∗05, similar to that in MR1∗04 heterozygous individuals (18).

MR1 expression is severely reduced during infection with HSV-1, and this virus-induced effect was observed for all MR1 allomorphs studied here. However, the viral impairment is selective for nascent, ER-resident MR1 molecules that are not yet Ag-loaded. At homeostasis, the half-life of MR1 molecules expressed on the cell surface and bound to Ag is determined by a combination of natural ligand dissociation, endocytosis and lysosomal degradation (45, 81, 82). During HSV-1 infection, this half-life is impacted by impaired endocytosis (53). Hence, the addition of ligand to the MR1 allomorphs prior to HSV-1 infection completely rescued cell surface MR1 allomorph expression for all except the MR1∗04:01 and MR1∗04:02 molecules, despite the significant reduction of overall MR1 expression caused by viral infection (29, 53, 54). The continuous presence of vitamin B6-related ligands that are preferentially bound by MR1∗04 could explain the attenuated phenotype observed with HSV-1 infection of these allomorph cell lines (53). Furthermore, they may be differentially impacted by increased pyridoxal kinase expression during HSV-1 infection, which might reduce the availability of pyridoxal in the culture medium for capture by the MR1∗04 allomorphs (83, 84). Therefore, natural endogenous ligand availability only partially preserves cell surface expression of MR1∗04:01 and MR1∗04:02 molecules in the face of infection with HSV-1.

Taken as a whole, MR1 has a low level of polymorphism, and most alleles create MR1 allomorphs that are structurally and functionally very similar to the canonical MR1∗01. The exceptions are rare allomorphs MR1∗05, which behaves as a near null mutant, and MR1∗04:01 and MR1∗04:02, which select dramatically different ligands from the MR1∗01. These findings are consistent with the constrained T cell repertoire of MAIT cells and their MR1-restricted Ag recognition, suggesting strong evolutionary pressure for pattern-like recognition of the well-defined riboflavin metabolites, 5-OP-RU and 5-OE-RU.

## Experimental procedures

### Human ethics

PBMCs were isolated from blood donations as previously described (85). Blood was sourced from the Australian Red Cross Service (authorized by the Australian Red Cross Blood Service Material Supply Agreement with the University of Melbourne and approved by the University of Melbourne Human ethics committee; ID 28023 and 12540). All human studies conducted abide by the Declaration of Helsinki principles.

### MR1 ligands and cell culture media

Ac-6-FP (Schircks Laboratories) was dissolved at 5 mM in water and supplemented with 17 mM NaOH. 5-OP-RU was synthesized in house as previously described (86) and stored as a stock solution at 5 mM in DMSO. HMB (Sigma Aldrich, Cat. No. 146862) was stored in DMSO as an 80 mM stock solution. Pyridoxine hydrochloride (Sigma-Aldrich, Cat. No. 58–56–0) was dissolved in water and stored as a stock solution of 100 mM. For cellular assays, all ligands were diluted in supplemented RPMI-1640 (Gibco, 11875093) or supplemented RPMI without vitamin B6, glutamine or hydroxyproline, made in-house. For HSV-1 infection assays, cells were cultured in Dulbecco’s Modified Eagle’s Medium (DMEM, Gibco, Cat. No. 11995065) supplemented with 10% fetal bovine serum (FBS, Bovogen Cat. No. 2207C).

### Generation of reporter cell lines (C1R and 293T derivatives)

To produce MR1 allelic variants (MR1∗02, MR1∗03, MR1∗04:02, MR1∗06), single and double mutants for MR1∗05 and artificial mutants of His90 and Asp115, a modified version of pMSCV-IRES-eGFP (pMIG-II (87)) previously cloned with the protein coding region of the MR1∗01, MR1∗04:01 or MR1∗05 gene (21, 40) was subjected to site directed mutagenesis using the Agilent QuikChange II kit (Cat. No. 200524) according to the manufacturers’ protocol. Mutagenesis primers were designed using the Agilent Quik Change Primer Design tool. To produce MR1∗05 allele, comprising three separate mutations, a synthetic gene string encoding the MR1∗05 protein coding sequence (Integrated DNA Technologies) was cloned into the pMIG-II vector. All newly generated gene construct sequences were verified by Sanger sequencing (Australian Genome Research Facility) prior to use. MR1 overexpressing cell lines were generated using retroviral transduction of MR1^null^ cell lines (40, 41) as previously described (88). MR1 was detected on the cell surface by staining with an anti-human MR1-PE antibody (clone 26.5, Biolegend Cat. No. 361106). As the pMIG-II vector encodes a green fluorescent protein (GFP) reporter, expressed 1:1 with the target gene, MR1 cell lines were bulk sorted to obtain cells matched for similar levels of GFP expression in order to reflect comparable MR1 gene expression. All data relating to MR1 expression in cell line assays have been GFP-matched using a gating strategy outlined in Figure S1*H*, to further match gene expression.

### Production of soluble allelic variants of MR1

DNA constructs encoding the heavy chains for each MR1 allomorph, β2m and the α and β chains of A-F7 and BV6-4, were transformed and overexpressed in *Escherichia coli* BL21. The MR1 and β2m inclusion bodies were refolded at a 2:1 ratio in the presence of HMB, Ac-6-FP and 5-OP-RU in 100 mM Tris-HCl pH 8.0, 2 mM ethylenediaminetetraacetic acid (EDTA), 5 M urea, 0.4 M arginine-HCl, 0.5 mM oxidized glutathione, 5 M reduced glutathione and 0.1 M phenylmethylsulfonyl fluoride. The MAIT TCRα and β chains were folded at 3:2 ratio in a similar refold buffer as described above. The MR1-β2m-ligand complexes and MAIT TCR complexes were then dialyzed three times against 10 mM Tris pH 8.0 at 4 °C for 16 h. The dialysate was purified by three consecutive stages: a crude anion exchange using DEAE Sepharose resin, Superdex 200 10/300 size exclusion chromatography and HiTrap-Q HP anion exchange. The purified MR1-β2m-ligand complexes and MAIT TCRs are then snap-frozen in liquid nitrogen before being stored at −80 °C.

### Thermal shift assay

To investigate the stability of the different MR1 allomorphs bound to either Ac-6-FP, HMB or 5-OP-RU, the purified MR1-ligand complexes were subject to a thermal shift assay using a real-time detection system (Rotor-Gene Q). Purified MR1-ligand proteins were incubated with a fluorescent SYPRO Orange dye heated at a rate of 1 °C/min until the temperature reached 95 °C. The fluorescence emission of the SPYRO Orange dye was monitored (excitation at 530 nm and emission at 610 nm) and used to determine the thermal melting temperature of the different MR1 proteins. Data are representative of n = 3 independent experiments conducted, with technical triplicates performed.

### Crystallization and structural determination

For the MR1 allomorph structures, purified MR1- β2m-ligand complexes were mixed and incubated with A-F7 in a 1:1 molar ratio for 20 min at 4 °C and prepared at a final concentration of 3 to 5 mg/ml. The ternary complexes for MR1∗02, MR1∗03 and MR1∗05 were crystallized using hanging drop vapor diffusion with a liquid reservoir consisting of 100 mM Bis-Tris propane (pH 6.1–6.7), 12 to 18% w/v PEG3350, and 200 mM sodium acetate. The empty MR1∗04 complex was crystallized in 20% PEG6000, 0.1 M HEPES pH 7.2, 0.1 M lithium chloride. The crystals were then washed in reservoir solutions supplemented with 15% glycerol for cryoprotection prior to flash-cooling in liquid nitrogen. Datasets were collected on the Australian synchrotron MX2 beamlines at 100K for the MR1∗02, MR1∗03, MR1∗04 and MR1∗05 ternary complexes at a resolution of 2.2, 2.1, 2.8 and 2.2 Å, respectively. The diffraction datasets were then reprocessed with XDS (89) followed by scaling and merging with Aimless from the CCP4 software suite (90). The MR1∗01-5-OP-RU complex (PDB accession code 6PUC) without the ligand was used as a search model for molecular replacement. The ternary complex models were built in COOT (91), and numerous cycles of refinement were carried out using phenix.refine (7). The data collection statistics and crystallographic data of the final MR1-TCR models are summarized in Table S2.

### Surface plasmon resonance

Biotinylated MR1 allomorphs were purified in the presence of 5-OP-RU, with the exception of MR1∗04 which was biotinylated in an unliganded state. The biotinylated MR1 complexes were coupled to a streptavidin sensor chip at 2500 response units. Purified MAIT TCRs, A-F7 and BV6-4, flowed over the chip at concentrations of up to 80 μM. The SPR experiment was conducted on BIAcore T200 instrument using 10 mM HEPES pH 8.0, 150 mM NaCl, 0.005% P20 surfactant at a flow rate of 5 μl per minute at a temperature of 25 °C. The equilibrium dissociation constants (K_D_) were calculated at steady state affinity analysis with the non-linear curves fitted using GraphPad Prism assuming 1:1 binding. All titrations were repeated in duplicate for two independent experiments.

### MR1 surface staining

For MR1 surface expression assays, Cell Trace Violet (CTV) labelling was used to multiplex different cell lines into a single well of a culture plate. C1R cell lines were stained with either 2 μM, 0.2 μM, 0 μM CTV (Invitrogen, Cat. No. C34557) diluted in PBS for 20 min at room temperature. CTV-labelled cells were washed twice with PBS to remove excess CTV and mixed together at a 1:1:1 ratio. The cell line mixture was plated at 1.5 × 10^5^ total cells per well in a 96-well U-bottom tissue culture (TC) microplate (Corning Product No.3799) before the addition of MR1 ligands. For ligand-dependent MR1-upregulation assays, titrating doses of 5-OP-RU, Ac-6-FP and pyridoxal were added to cells before 16-h overnight incubation. For time course MR1-upregulation assays, a single dose of either 5-OP-RU, Ac-6-FP, pyridoxal or HMB was added at 1, 2, 4, 8, 16 and 24 h prior to assessing MR1 expression. After incubation, cells were washed with PBS and stained with fixable viability dye eFluor 780 (eBioscience, Cat. No. 65-0865-18) and MR1 PE for 25 min at room temperature. Cells were washed twice and resuspended in PBS before acquisition on a LSR Fortessa (BD Biosciences). Data acquisition and analysis were carried out using FACSDiva (BD Biosciences) and FlowJo version 10.9.0 (BD Biosciences).

For the evaluation of MR1 surface expression in HSV-1 infected cells, 1.5 × 10^5^ 293T cells of each allele or MR1^null^ were plated in separate wells of a 24-well flat bottom TC plate (Corning, Cat. No. CLS3516), 24 h before infection. Cells were optionally treated with 5 μM Ac-6-FP at time of plating, and not replaced in the media after infection, or added for the final 4 h of the 20-h infection. Cells were harvested by trypsinisation, washed with PBS and stained with UV blue fixable viability dye (ThermoFisher Scientific, Cat. No. L23105) and then MR1-PE (clone 26.5, BioLegend, Cat. No. 361106) for 25 min on ice. Cells were fixed (BD Biosciences Cat. No. 554655) before acquisition on an LSR-II (BD Biosciences). Data acquisition and analysis were carried out using FACSDiva (BD Biosciences) and FlowJo version 10.8.1 (BD Biosciences).

### MR1 intracellular staining

To assess intracellular expression of MR1, 10^5^ MR1 overexpressing, MR1^null^ or parental C1R cells were seeded into a 96-well V-bottom microplate (Merck, Cat. No. M1561), washed with PBS and were stained with fixable viability dye eFluor 780 for 20 min. Without washing, cells were fixed in 2% PFA for 20 min at room temperature. Cells were washed once with PBS and then stained with MR1 PE in 0.3% Saponin (Sigma-Aldrich, Cat. No. 8047-15-2) overnight at 4 °C. Cells were washed and resuspended in PBS before acquisition on a LSR Fortessa. Data acquisition and analysis were carried out using FACSDiva and FlowJo version 10.9.0.

### MR1 immunoblotting and immunoprecipitation

Immunoblotting and immunoprecipitation for the detection of MR1 were performed as described previously (14, 45). In brief, 2 × 10^6^ MR1 overexpressing, MR1^null^ or parental C1R cells were washed three times with PBS before lysis with 0.5% IGEPAL CA-630 (Sigma-Aldrich, Cat. No. I8896), 50 mM Tris-HCl (pH 7.4), 5 mM MgCl_2_ with Protease Inhibitor Cocktail (Cell Signalling Technologies Cat No. 5871). Lysate was centrifuged at 13000*g* to separate the nuclear fraction, and total protein was quantified using Pierce BCA protein assay kit (Thermo Scientific, 23225). For immunoprecipitation, lysates were precleared with Protein G Sepharose (Millipore, Cat. No. P3296) and MR1 was precipitated using Protein G Sepharose with an anti-MR1 antibody targeting the final 15 residues of the human MR1 CT (14) or anti-MR1-8F2.F9 (46). Proteins were denatured using LDS sample buffer (Invitrogen, Cat. No. NP0007) and 100 mM DTT at 72 °C for 10 min and separated on Bolt Bis-Tris Plus Mini Protein Gels, 4 to 12% (Invitrogen, Cat. No. NW04120BOX). Proteins were immunoblotted onto Iblot 2 Transfer Stacks nitrocellulose membranes (Thermo Scientific, Cat. No. IB23001) using the iBlot 2 dry blotting system (Invitrogen, Cat. No. IB21001). MR1 was detected using anti-MR1-CT. Control actin and tubulin were detected using clone C4 and DM1A (Sigma-Aldrich Cat No. MAB1501 and Abcam Cat No. ab7291). Secondary anti-Rabbit-horseradish peroxidase (HRP) (Amersham Cat. No NA934V) and anti-Mouse HRP (Amersham Cat. No. NA931V) were used to detect primary antibody stain. Immobilon Forte western HRP substrate (Millipore, Cat. No. WBLUF) was used to visualize bands, which were detected on a ChemiDoc imaging system (Biorad, Cat. No. 12003153).

For the evaluation of total MR1 expression in HSV-1 infected cells, 3 × 10^5^ 293T cells of each allele or MR1^null^ were plated in separate wells of a 12-well flat bottom TC plate (Corning, Cat. No. CLS3513), 24 h before infection. Cells were treated with 5 μM Ac-6-FP either at time of plating, and not replaced in the media after infection, or added for the final 4 h of the 22-h infection. Cells were released from the plate by scraping and lysed in cell lysis buffer (50 mM NaCl, 50 mM TRIS pH8, 1% IGEPAL, 1% Triton X-100) supplemented with protease inhibitor cocktail (Sigma). Proteins were denatured using sample buffer (BioRad, Cat. No. 1610791) and reducing agent (BioRad, Cat. No. 1610792), separated on Criterion XT Precast Gel 4 to 12% (BioRad, Cat. No. 3450124) and transferred to PVDF membranes using the semi-dry Transblot Turbo transfer system (BioRad). MR1 was detected using anti-MR1-CT antibody with GAPDH (ThermoFisher Scientific, Cat. No. TAB1001) detection acting as loading control.

### Magnetic enrichment of TRAV1-2+ cells from PBMCs

Enrichment of TRAV1-2+ cells has been described previously (40, 92). In brief, 1.5 × 10^8^ PBMCs were stained with anti-TRAV1-2 PE (Biolegend, Cat. No. 351702 3C10) in magnetic-activated cell sorting (MACS) buffer (0.5% FBS, 2 mM EDTA in PBS) for 25 min at 4 °C. Cells were washed once with cold MACS buffer and incubated with anti-PE beads (Miltenyi Biotec Cat. No. 130-097-054) in MACS buffer for 20 min at 4 °C. Cells were washed with MACS buffer, resuspended and passed through LS column (Miltenyi Biotec, Cat. No. 130-042-401) while under magnetic duress. TRAV1-2+ cells are eluted from the LS column and resuspended in supplemented RPMI-1640.

### Intracellular staining of enriched T cells stimulated with allomorph cell lines

Stimulation of enriched T cells with C1R cell lines has been described previously (40). In brief, MR1 overexpressing, MR1^null^ or parental C1R cells were pulsed with titrating amounts of 5-OP-RU for 2 h and washed three times to remove any extracellular ligand in order to minimize T cell auto-presentation. C1R cells were resuspended in supplemented RPMI-1640 media and cultured at a 1:1 ratio with enriched TRAV1-2+ cells for a total of 6 h in a TC incubator. Brefeldin A (Sigma-Aldrich, Cat. No. 20350-15-6) diluted 1:1000 was added after 1 h of culture. Following the culture, cells were stained with a fixable viability dye eFluor 780 for 10 min at room temperature. Cells were washed with PBS and then stained for 25 min at 4 °C with anti-CD3-BUV395 (BD Horizon, clone UCHT1, Cat. No. 563546), anti-CD4-BUV496 (BD Horizon, clone GK1.5, Cat. No. 612952), anti-CD8α-BUV805 (BD Horizon, clone SK1, Cat. No. 564912), anti-CD161-PE-Vio770 (Miltenyi Biotec, Clone REA631, Cat. No. 130-113-597), anti-TRAV1-2-PE, anti-CD19-APC-H7 (BD Pharmingen, Clone HIB19, 560727) and anti-CD69-APC (BD Pharmingen, clone FN50, Cat. No. 560967). Without washing, cells were fixed with 2% PFA in PBS for 20 min at room temperature. Cells were washed with PBS and stained intracellularly with anti-TNF-BV421 (BD Horizon, clone Mab11, Cat. No. 562783), anti-IFNγ-BV650 (BD Horizon, clone 4S.B3, Cat. No. 563416), and anti-IL-17A-PE-Dazzle 594 (Biolegend, clone BL168, Cat. No. 512336) in 0.3% Saponin overnight at 4 °C. The following morning, cells are washed with PBS before acquisition on a LSR Fortessa. Data acquisition and analysis were carried out using FACSDiva and FlowJo version 10.9.0.

### Production of MR1 tetramers

Refolding of MR1 and β2m inclusion bodies in the presence of ligand has been described previously (2). Briefly, a chemically defined refold buffer consisting of 100 mM Tris pH 8 (Sigma-Aldrich, Cat. No. T1503), 2 mM EDTA (Sigma-Aldrich, Cat. No. E5134), 400 mM L-Arginine monohydrochloride (Sigma-Aldrich, Cat. No. A5131), 0.5 mM L-Glutathione oxidized (Sigma-Aldrich, G4376), and 5 mM L-Glutathione reduced (Sigma-Aldrich, Cat. No. G4251) was prepared and cooled to 4 °C. 112 mg of hMR1∗04:01 modified with a C-terminal Cys and 56 mg of hβ2m inclusion bodies were added to 800 ml of refold buffer in the presence of 20 μM pyridoxal or other individual ligands overnight. The refold mixture was transferred into dialysis tubes (Repligen, Cat. No. 132660) and dialyzed against 10 mM Tris, pH 8 overnight. The refolded protein was purified using fast protein liquid chromatography (FPLC) sequentially *via* ion exchange with DEAE Sepharose beads (Cytiva, Cat. No. 17070901), size exclusion chromatography with the Superdex-75 column (Cytiva, Cat. No. 17517401), and finally by the high-performance ion exchange MonoQ column (Cytiva, Cat. No. 17516601). The resulting purified monomers were biotinylated using an EZ-link maleimide-PEG2-biotin kit (Thermo Scientific, Cat. No. A39261) and purified using a MonoQ column. Tetramerization was achieved by mixing 5 μg of biotinylated monomers with 1 μg of streptavidin-PE (BD Pharmingen, Cat. No. 554061) in five additions with 10 min of incubation time at room temperature in between each addition.

### Magnetic enrichment and expansion of MR1∗04:01-pyridoxal tetramer positive cells from PBMCs

PBMCs were stained with MR1∗04:01-pyridoxal tetramer PE at a final concentration of 25 μg/ml. Magnetic enrichment of MR1∗04:01-pyridoxal tetramer + cells was performed as described earlier. The enriched fraction was stained for 20 min on ice with fixable viability dye eFluor 780, anti-CD3 BUV395, anti-CD4 BUV496, anti-CD8α BUV805, anti-TRAV1-2 APC (Biolegend, clone 3C10, Cat. No. 351708), and anti-CD161 PE-Vio770. CD3+CD8+MR1∗04:01-pyridoxal tetramer + cells were sorted on the BD FACSAria III cell sorter. The sorted cells were then stimulated in a 96-well round bottom TC microplate using plate-bound anti-CD3 (BD, clone UCHT1, Cat. No. 550368) and anti-CD28 (BD, clone L293, Cat. No. 348040) antibodies in combination with soluble recombinant 200 U/ml human IL-2 (Peprotech, Cat. No. 200-02), 25 ng/ml human IL-7 (Peprotech, Cat. No. 200-07), 5 ng/ml human IL-15 (Peprotech, Cat. No. 200-15), and 1 μg/ml phytohemagglutinin-L (PHA-L) (Roche, Cat. No. 11249738001) for 3 days. Expanding cells were maintained in media consisting of human IL-2, IL-7, and IL-15 for 2 weeks before use in cellular assays.

### HSV infection of MR1 allomorph cell lines

After first replacing the FBS supplemented DMEM media, cells were infected for the 1 h period of adsorption (with gentle rocking at 37 °C) with HSV-1 strain F, at a multiplicity of infection of 2, or mock-infected. Cells were again washed before continuing with the assay. HSV-1 virus was grown and titrated on Vero cells.

### Cellular and statistical analysis

Flow cytometric data were acquired on a LSR Fortessa X20 with UV upgrade or LSR-II (BD Biosciences), and data acquisition and analysis were carried out using FACSDiva and FlowJo version 10.8.1 or 10.10.0 (BD Biosciences) respectively. Graphs of flow cytometric data and statistical analysis were performed on Prism 10 (Graphpad). Statistical significance was determined using a two-way ANOVA with Sidak’s multiple comparison test, paired or unpaired Students *t* test.

## Data availability

The coordinates of the MAIT A-F7 TCR in complexes with MR1∗02-5-OP-RU, MR1∗03-5-OP-RU, MR1∗04-empty and MR1∗05-5-OP-RU have been deposited in the Protein Data Bank under accession codes 9YOT, 9YOU, 9YOV and 9YOW respectively.

## Supporting information

This article contains supporting information (18, 35).

## Conflict of interests

The authors declare the following financial interests/personal relationships which may be considered as potential competing interests: A. J. C., Z. C., J. R. and J. M. are inventors on university-owned patent rights (patent families WO/2015/149130 and WO/2014/005194) describing MR1 tetramers, licensed for commercial use to Immudex and for nonprofit use to NIH Tetramer Core Facility, and describing MR1 ligands. The other authors declare no competing interests.

## Supporting information

Nelson-et-al
