# Supplementary material for: Allelic variation alters expression and antigen presentation of MR1 allomorphs
Source: J Biol Chem. 2026 Apr 17;302(6):111470. doi: 10.1016/j.jbc.2026.111470 (PMC13213668; doi:10.1016/j.jbc.2026.111470)
Supplement: Nelson-et-al [file mmc1.docx]

**Supplementary Table 1. Summary of the allelic variants of MR1**

| **MR1 allele** | **Nucleotide mutation** | **Protein Mutation** | **Domain** | **Reported Frequency** |
| --- | --- | --- | --- | --- |
| **MR1*01** | N/A |  |  | **71.4%^(1)^** |
| **MR1*02** | CAT>CGT | H>R (P17) | α1 domain | **25%**^(1)^ |
| **MR1*03** | ATC>GTC | I>V (P121) | α2 domain | **0.9%**^(1)^ |
| **MR1*04:01** | CGC>CAC | R>H (P9) | α1 domain | **0.9%**^(1, 2)^ |
| **MR1*04:02** | CGC>CAC  CAT>CGT | H>R (P17)  R>H (P9) | α1 domain |  |
| **MR1*05** | GAG>GGG  CAC>CAA  ATT>GTT | E>G (P52)  H>Q (P90)  I>V (P244) | α1, α2 and α3 domain | **0.9%**^(1)^ |
| **MR1*06** | AGG>AAG | R>K (P304) | Cytoplasmic Tail | **0.9%**^(1)^ |

**Supplementary Table 2. Data collection and refinement statistics.**

|  | AF7 TCR-MR1*02 5-OP-RU | AF7 TCR-MR1*03 5-OP-RU | AF7 TCR-MR1*04 empty | AF7 TCR-MR1*05 5-OP-RU |
| --- | --- | --- | --- | --- |
| Wavelength | 0.9537 | 0.9537 | 0.9537 | 0.9537 |
| Resolution range | 46.62 - 2.20 (2.22 - 2.20) | 47.79 - 2.10 (2.12 - 2.10) | 43.44 - 2.80 (2.83 - 2.80) | 47.35 - 2.30 (2.33 - 2.30) |
| Space group | C 1 2 1 | C 1 2 1 | P 1 21 1 | C 1 2 1 |
| Unit cell | 218.226 71.054 144.176 90 104.57 90 | 215.964 69.919 142.677 90 104.33 90 | 62.28 140.788 114.831 90 89.97 90 | 213.432 69.501 143.205 90 103.94 90 |
| Total reflections | 418388 (20525) | 408203 (21281) | 217778 (34387) | 315716 (15388) |
| Unique reflections | 107970 (5263) | 119799 (5945) | 61558 (9339) | 90694 (4497) |
| Multiplicity | 3.9 (3.9) | 3.4 (3.6) | 3.5 (3.7) | 3.5 (3.4) |
| Completeness (%) | 99.4 (99.1) | 99.4 (99.9) | 98.01 (96.79) | 99.8 (99.9) |
| Mean I/sigma(I) | 8.9 (1.5) | 11.3 (3.1) | 10.0 (2.1) | 10.9 (1.3) |
| Wilson B-factor | 41.66 | 37.40 | 55.24 | 45.07 |
| R-merge | 0.093 (0.976) | 0.055 (0.401) | 0.086 (0.679) | 0.075 (0.964) |
| R-meas | 0.108 (1.132) | 0.066 (0.471) | 0.258 (1.449) | 0.088 (1.145) |
| R-pim | 0.055 (0.567) | 0.035 (0.246) | 0.050 (0.389) | 0.047 (0.612) |
| CC1/2 | 0.997 (0.698) | 0.998 (0.918) | 0.997 (0.843) | 0.998 (0.485) |
| R-work | 0.1847 (0.2901) | 0.1601 (0.2330) | 0.2109 (0.3410) | 0.1806 (0.3550) |
| R-free | 0.2265 (0.3466) | 0.1916 (0.2632) | 0.2479 (0.3711) | 0.2118 (0.4020) |
| Number of non-hydrogen atoms | 13832 | 14202 | 13012 | 13762 |
| macromolecules | 12928 | 13022 | 12747 | 12928 |
| ligands | 63 | 104 | 18 | 64 |
| solvent | 841 | 1076 | 247 | 770 |
| Protein residues | 1612 | 1615 | 1613 | 1607 |
| RMS(bonds) | 0.003 | 0.004 | 0.002 | 0.002 |
| RMS(angles) | 0.61 | 0.67 | 0.50 | 0.55 |
| Ramachandran favoured (%) | 97.80 | 98.43 | 96.41 | 98.10 |
| Ramachandran allowed (%) | 2.20 | 1.57 | 3.59 | 1.90 |
| Ramachandran outliers (%) | 0.00 | 0.00 | 0.00 | 0.00 |
| Rotamer outliers (%) | 1.31 | 1.08 | 3.27 | 1.59 |
| Average B-factor | 51.76 | 46.23 | 59.68 | 51.72 |
| macromolecules | 51.69 | 46.07 | 59.89 | 51.80 |
| ligands | 43.21 | 41.56 | 64.88 | 40.73 |
| solvent | 53.46 | 48.62 | 48.58 | 51.25 |

Statistics for the highest-resolution shell are shown in parentheses.

**
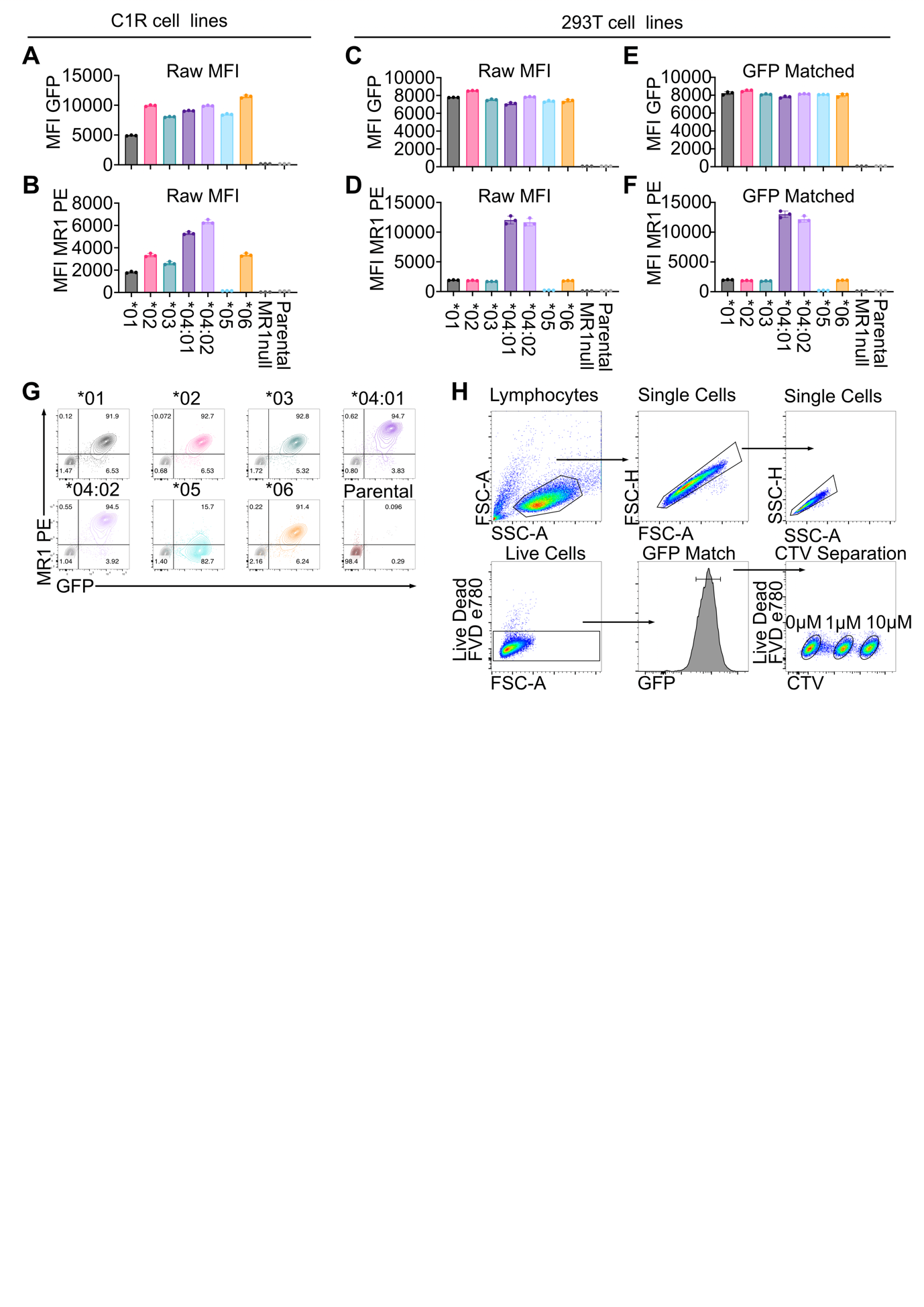
**

**Supplementary Figure 1. Gating strategy and characterisation of MR1 allomorph cell lines**

**(A-D)** Constitutive expression of GFP (top panels) and median fluorescence intensity (MFI) from anti-MR1-PE surface staining (bottom panels) for C1R MR1 allomorphs cell lines (**A and B**) or 293T MR1 allomorph cell lines (**C and D**). (**E and F)** Constitutive expression of GFP (**E**) and median fluorescence intensity (MFI) from anti-MR1-PE surface staining (**F**) for 293T MR1 allomorph cell lines matched for GFP/MR1 expression. **(G)** Representative flow cytometry panels showing 293T.MR1^null^ cells transduced with MR1*01 (black), MR1*02 (pink), MR1*03 (green), MR1*04:01 (dark purple), MR1*04:02 (light purple), MR1*05 (blue), MR1*06 (orange) and parental 293T cells (dark red) compared to 293T.MR1^null^ cells (grey) for anti-MR1-PE surface staining (y axis) and GFP (x axis). Quadrant gates display percentage of live cells. **(H)** MR1 allomorph C1R or 293T cell lines were gated based on size (forward scatter area vs side scatter area) and doublets removed using forward and side scatter (height vs area). Live cells were identified as fixable viability dye eFluor 780 (Live Dead FVD e780) negative cells. Live cells were gated to match GFP expression and separated using gates corresponding to titrating amounts of cell trace violet (CTV) (0 µM, 1 µM and 10 µM). Data are from one experiment performed in triplicate. Bars represent mean and error bars are ± standard deviation.

**
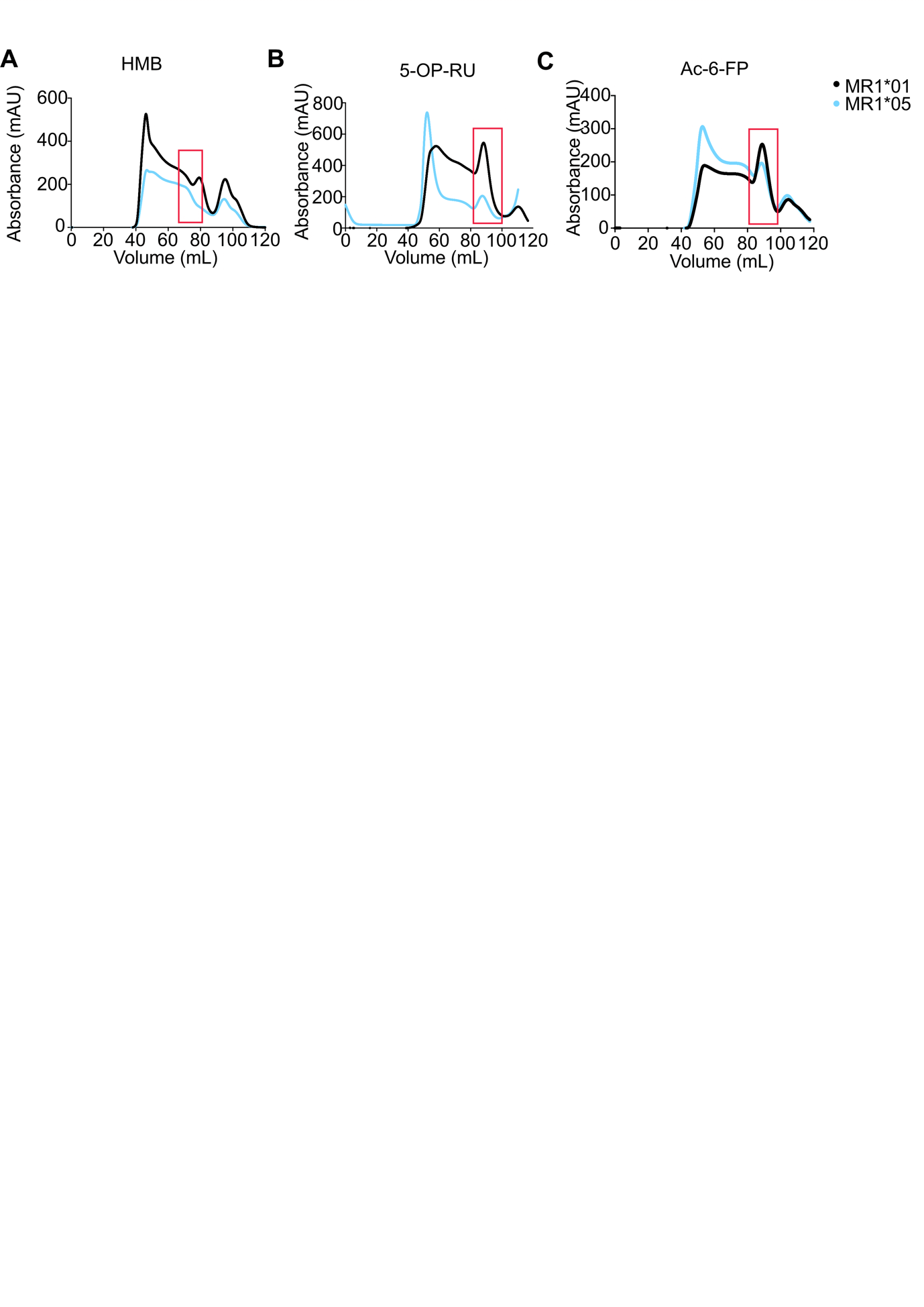
**

**Supplementary Figure 2. MR1*05 allelic variants show differential refolding efficiencies in response to various MR1 ligands**

Size exclusion chromatogram of the MR1*01-β2m (in black) and MR1*05-β2m (in light blue) refolded with **(A)** HMB, **(B)** 5-OP-RU and **(C)** Ac-6-FP. Upon successful refolding, MR1-β2m-ligand complex elutes at approximately 85 mL, as depicted by a peak highlighted by the red box. MR1*05-β2m refolded in the presence of 5-OP-RU and Ac-6-FP, while it did not refold in the presence of the less potent HMB ligand.

**Supplementary Figure 3. Alignment of MR1 amino acid sequence across species**

MR1 amino acid sequences for *Homo sapiens* (human, MR1*01 sequence), *Mus musculus* (mouse), *Rattus norvegicus* (rat), *Sus scrofa* (pig), *Bos taurus* (bovine), *Ovis aries* (sheep) and *Sacrophillus harrisii* (Tasmanian devil) were aligned using CLC sequence viewer 8 (Qiagen). Sequences were annotated for α1, α2, α3, transmembrane and intracellular domains. Locations of allomorph mutations are annotated with red arrows highlighting the mutated residues.

**
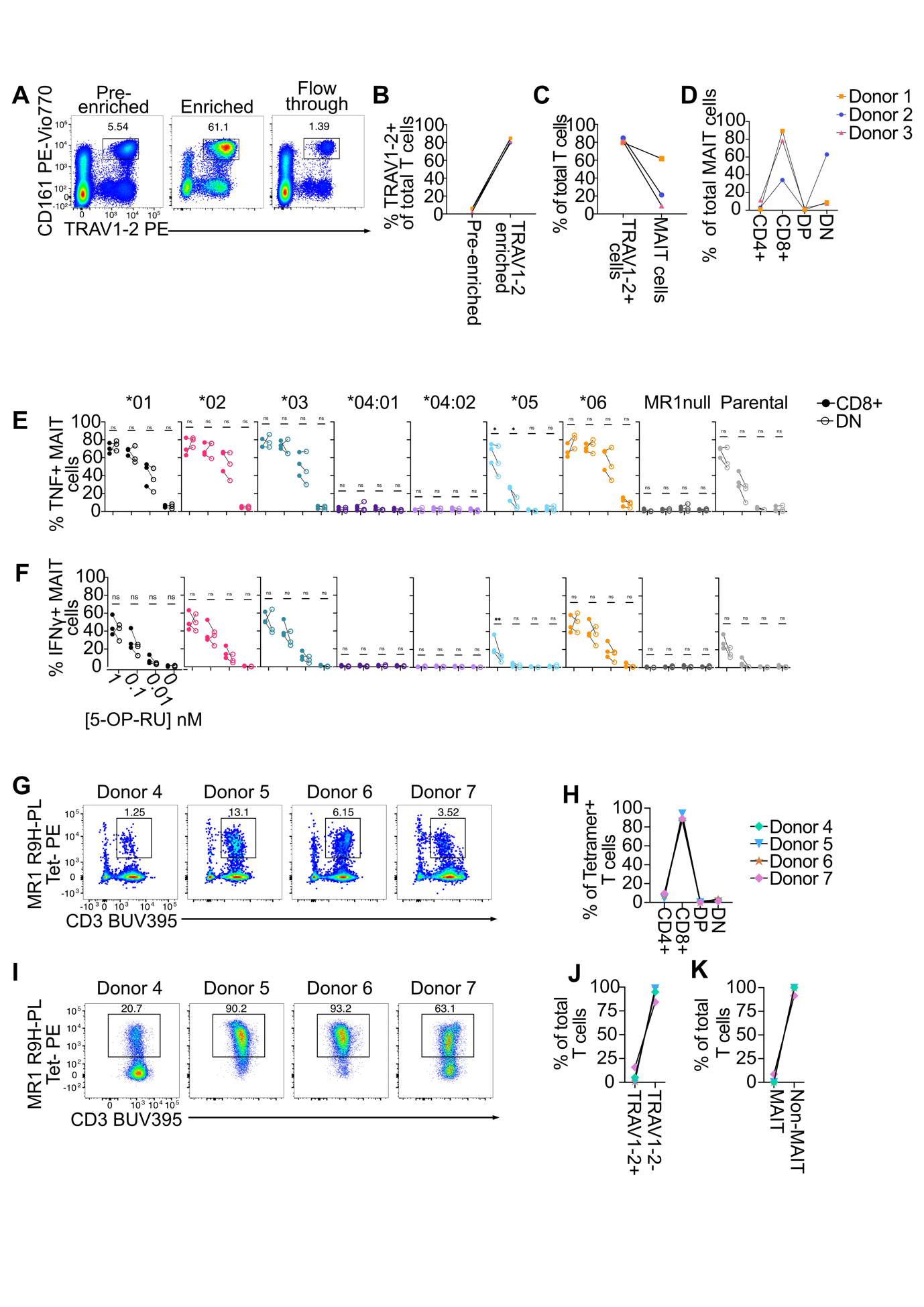
**

**Supplementary Figure 4. Characterisation of MAIT and diverse T cell responses to MR1 allomorph cell lines**

**(A)** Representative flow cytometry panels displaying MAIT cells from PBMCs identified using anti-CD161-PE-Vio770 (y axis) and anti-TRAV1-2-PE (x axis) prior to anti-PE microbead enrichment (left panel), post enrichment (centre panel) and in the flow through post enrichment (right panel). **(B)** Paired graph of the percentage of TRAV1-2+ cells of total CD3+ cells prior to and post enrichment from each PBMC donor. **(C)** Paired graph of the percentage of TRAV1-2+ cells and MAIT (TRAV1-2+CD161+) cells from total CD3+ cells in each enriched sample. **(D)** Paired graph of the coreceptor expression from MAIT cells in each donor. (**E-F)** Percentage of TNF (**E**) or IFNγ (**F**) positive MAIT cells separated based on coreceptor expression (CD8 positive closed circles and double negative open circles) in response to MR1 allomorph cell lines pulsed with titrating 5-OP-RU or in the absence of ligand. Data are from one experiment using three healthy blood donors with symbols representing individual donors. Statistical significance was determined using a two-way ANOVA with Sidak’s multiple comparison test with ** (p<0.01), * (p<0.05) or ns (≥0.05). **(G)** Representative flow cytometry panels of enriched MR1^R9H^-pyridoxal (PL) tetramer-PE positive cells from four additional donors, displaying MR1^R9H^-PL tetramer-PE (y axis) and anti-CD3-BUV395 (x axis). **(H)** Paired graph of coreceptor usage from tetramer-enriched T cells. **(I)** Representative flow cytometry panels of expanded T cells stained as above. **(J)** Paired graph showing percentage of TRAV1-2+ and TRAV1-2- cells from expanded T cells. **(K)** Paired graph showing percentage of MAIT (TRAV1-2+ CD161+) and non-MAIT (TRAV1-2 single positive, CD161 single positive or double negative) cells from total *in vitro* expanded T cells. Data are from one experiment using four healthy blood donors. Symbols represent individual donors. Statistical significance was determined using a two-way ANOVA with Sidak’s multiple comparison test.

**References for Supporting Information**

1. E. Rozemuller *et al.*, MR1 encompasses at least six allele groups with coding region alterations. *HLA* **98**, 509-516 (2021).

2. L. J. Howson *et al.*, Absence of mucosal-associated invariant T cells in a person with a homozygous point mutation in MR1. *Science Immunology* **5**, eabc9492 (2020).
